# Evaluation of Three Sample Preparation Methods for LC-HRMS Suspect Screening of Contaminants of Emerging Concern in Effluent Wastewater

**DOI:** 10.1021/acs.analchem.5c01659

**Published:** 2025-11-19

**Authors:** Dana Orlando-Véliz, Rocío Inés Bonansea, Manuel García-Vara, Varvara Nikolopoulou, Miren López de Alda

**Affiliations:** † Water, Environmental and Food Chemistry Research Unit, Dep. of Environmental Chemistry, IDAEA-CSIC, 203229 Institute for Environmental Assessment and Water Research (IDAEA-CSIC), C/Jordi Girona 18−26, 08034 Barcelona, Spain; ‡ PhD student in the Analytical and Environmental Chemistry PhD Program at the University of Barcelona, C/Martí i Franquès, 1-11, Les Corts, 08028 Barcelona, Spain; § Department of Environmental Health Sciences, Yale School of Public Health, Yale University, New Haven, Connecticut 06510, United States

## Abstract

Suspect screening is a valuable tool for studying the pollution footprint of environmental samples, but the sample preparation (SP) method significantly affects the results. In this study, three SP methods—lyophilization, direct injection, and online solid-phase extraction (SPE)—were evaluated for suspect screening of contaminants of emerging concern (CECs) in effluent wastewater using liquid chromatography-high resolution mass spectrometry (LC-HRMS). LC-HRMS was performed with a Q-ToF mass analyzer in both positive and negative electrospray ionization modes, and pollutants were identified using the NORMAN SusDat database. A total of 119 CECs were identified with high confidence (all above level 2a). Of these, 115 were detected using lyophilization, 37 with direct injection, and 49 with online SPE. Principal component analysis revealed distinct patterns for each SP method. In all cases, the pollution profile was dominated by pharmaceuticals, followed by industrial chemicals. Articaine, a local anesthetic previously unreported in the aquatic environment, was the most abundant compound found. Industrial chemicals included phthalates, flame retardants, benzotriazoles, and perfluoroalkyl substances, among others. The remaining identified CECs were personal care products, pesticides, and drugs of abuse. Compound prioritization, based on abundance, persistence, mobility, bioaccumulation potential, and toxicity, identified the antibiotics clindamycin and tiamulin, the analgesic flufenamic acid, the musk fragrance metabolite galaxolidone, and the antidepressant venlafaxine metabolite, O-desmethyl venlafaxine, as top priority. These results highlight the importance of applying an appropriate SP method to obtain a comprehensive CEC footprint in suspect screening analysis and demonstrate the suitability of lyophilization for wastewater characterization.

## Graphical abstract

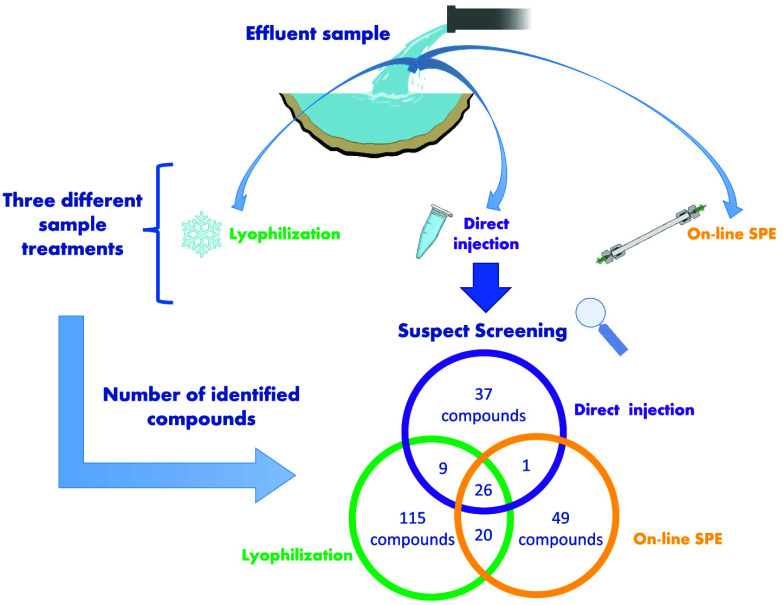

## Introduction

The exponential population growth and its associated economic activities have increased the water shortage globally, forcing authorities to implement various measures to preserve water quality.^1^ In Europe, wastewater reuse has become one of the main strategies to face this problem. Today, reclaimed water is present in many areas such as agricultural irrigation, aquifer recharge, landscaping, municipal sanitation, and even indirect potable water purification for human consumption.^2–4^ The minimum quality parameters required based on the intended use of treated water are compiled in the Regulation EU 2020/741.^5^ Although some wastewater treatment plants (WWTP) employ advanced remediation processes, they do not guarantee the complete removal of all organic micropollutants and this is strongly evident in the case of conventional treatment plants.^6–8^ As a result, these effluents become a source of pollutants emission into the environment.

These micropollutants are referred to as contaminants of emerging concern (CECs), chemical substances whose environmental fate and impact are not yet well characterized. The increasing environmental monitoring programmes worldwide and the improvement of the analytical methodologies and instrumentation in recent years have broadened the CECs chemical space.^4,8^ These compounds exhibit a wide range of physical-chemical properties (polarity, structure, solubility, etc.) and uses such as pharmaceuticals,^9–11^ personal care products,^12^ disinfection byproducts, pesticides,^13^ flame retardants,^14,15^ industrial chemicals,^16^ their derivatives, and other classes.^17,18^ To minimize the impact of CECs in the aquatic environment and ensure water sustainability, the EU Directive 2013/39 ^19^ established a list of priority substances to control chemical pollution in surface waters. Additionally, in 2015, it was introduced the concept of Watch List, which includes substances that may pose a significant risk to or via the aquatic environment.^20^ The purpose of this list is to monitor these substances and assess their potential risks in order to support future prioritization exercises.

Most CECs are not regulated or even known and, therefore, their discharge into the environment is not controlled. The inherent hazard associated with these pollutants is determined by their intrinsic physical-chemical properties, which influence their environmental risk and fate.^21^ CECs can be persistent in the environment, mobile in aqueous media, and exhibit toxicity or bioaccumulation in aquatic organisms. CECs can subsequently result in ecosystem deterioration and enter the human trophic chain becoming a human health risk.

Monitoring CECs is a valuable tool to control their environmental occurrence. However, the successful detection of some of these compounds can be highly challenging due to their diverse chemical properties and the complexity of environmental matrices. Targeted monitoring programs handle a very narrow range of CECs and, therefore, do not provide a complete characterization of the CEC footprint in the analyzed samples.

In the last decades, liquid chromatography coupled to high resolution mass spectrometry (LC-HRMS) has led to the development of suspect screening (SS) and nontarget screening (NTS) methodologies, which have significantly widened CEC surveillance. In both techniques, the range of compounds covered by the method is not known a priori. However, suspect-type analyses allow the investigation of so-called “known unknowns” by comparing acquired spectral features against suspect lists from databases.^22^ Depending on the parameters contrasted, i.e., exact mass match, isotopic pattern fit, coincidence and match of fragmentation profile, etc. the confidence level of identification achieved will be different.^23^ In this type of analyses, sample treatment becomes a critical step, as selective extraction and cleanup steps can result in the loss of many CECs while more universal techniques can be affected by high matrix effects and/or low sensitivity. In this context, optimizing sample preparation is essential for a holistic characterization of CECs.

Therefore, the objectives of this work were (i) to evaluate the efficiency of three different sample preparation approaches for CEC analysis in effluent wastewater samples using LC-HRMS suspect screening, (ii) to characterize the CECs present in the samples under study, and (iii) to prioritize the identified compounds based on their occurrence and potential environmental risk.

## Experimental Section

### Reagents and Chemicals

Information on the isotopically labeled compounds used as internal standards (IS) for data quality control is provided in Table S1 in Supporting Information. All solvents used were LC-HRMS grade. Acetonitrile (ACN), methanol (MeOH) and water used for LC-HRMS analysis were purchased from Thermo Fisher Scientific Inc. (Waltham, MA). MeOH, ethyl acetate (EtOAc) and water (H_2_O) used for sample preparation, and formic acid (purity >98%) and ammonium acetate used for chromatographic separation were purchased from Merck (Darmstadt, Germany).

### Case Study Area and Sample Collection

The WWTP under study is provided with conventional secondary treatment based on activated sludge. It is located in an industrial area in Barcelona province (Catalonia, Spain). This WWTP receives 40,000 m^3^/day of, mainly, industrial and urban wastewater. It has capacity for 300,000 inhabitants. From this WWTP, flow-proportional 24h composite effluent wastewater samples were collected in three different days (Wednesday, Friday and Sunday) within a week in January 2022. Two fractions of 400 mL of the 24h composite effluent samples were transferred to glass amber bottles and stored at −20 °C until analysis.

### Sample Preparation Methods

Three different sample preparation methods were evaluated for their efficiency in the nonselective extraction of CECs from wastewater effluent samples for subsequent wide scope suspect screening analysis. In all cases, before any further processing, samples (400 mL) were spiked with a mixture of isotopically labeled standard compounds at a concentration of 1 μg/L to monitor analytical performance and instrumental sensitivity.

Sample preparation method 1 (SP-1) is a modified version of an approach previously developed by our group.^6^ It involves freeze-drying the sample, followed by redissolution of the residue in a series of solvents with different polarities, which is, in principle, a simple and cost-effective sample preconcentration method.^13,24–26^ Specifically, in this method, 200 mL of the previously frozen sample was lyophilized using a LyoAlfa freeze-dryer (Telstar) at a final condenser temperature of −64 °C and a vacuum pressure of 0.031 mbar. This process requires 2–4 days to complete a batch of six samples. The residue was then reconstituted with 15 mL of MeOH, followed by 15 mL of EtOAc. The extracts were transferred to a glass centrifuge tube and centrifuged at 4000 rpm for 10 min (Eppendorf Centrifuge 5810R). The supernatant was then evaporated with a stream of N_2_ at 10 psi to a final volume of 0.2 mL and reconstituted to 1 mL with HPLC-grade water. Finally, the obtained extracts were centrifuged at 10,000 rpm for 10 min and transferred to HPLC vials for subsequent analysis by LC-HRMS.

In sample preparation method 2 (SP-2), 2 mL of the sample was centrifuged under the same conditions described above, and the supernatant was transferred to an HPLC vial for direct injection (100 μL) into the LC-HRMS system.

In sample preparation method 3 (SP-3), 2 mL of the sample was centrifuged and transferred to an HPLC vial as above and subjected to a subsequent online solid-phase extraction (online SPE) process. This process was performed using an Elute OLE (Online Extraction) system coupled to the LC-HRMS system and an ISOLUTE ENV+ online SPE cartridge (30 mm × 2.1 mm with a particle size of 40 μm) from Biotage (Uppsala, Sweden). The cartridge was first conditioned with 1 mL of ACN followed by 1 mL of H_2_O, both with a flow rate of 0.4 mL/min. Then, the water sample was loaded with 1.7 mL of a mixture of H_2_O/ACN at initial chromatographic conditions (95/5, v/v) at a flow rate of 0.3 mL min^–1^. A sample volume of 1.8 mL was loaded into the OLE system to fill the entire loop and 1 mL (full loop) of the sample was transferred to the chromatographic system. The ISOLUTE ENV+ online SPE cartridge is a polymeric cartridge that contains a hyper cross-linked polystyrene polymeric sorbent with a hydroxylated surface, designed for the extraction of compounds with a wide range of polarities from aqueous samples.

### Instrumental

LC-HRMS analysis was performed using an Elute UHPLC system coupled to an Impact II Q-TOF mass spectrometer (Bruker Daltonics, Billerica, MA) provided with a Vacuum Insulated Probe Heated Electrospray Ionization (VIP-HESI). For instrumental details, see Supporting Information SI 1. For data quality control, procedural blanks (HPLC water spiked with the IS mix) were prepared in the same way as samples, and analyzed together with the samples, solvent blanks, and standards.

### Postacquisition Data Processing and Confirmation Procedure

The retrospective analyses of the samples involved comparison with the suspect list mzCloud (S19, https://www.norman-network.com/nds/SLE/), which contains approximately 8,000 CECs from various applications. This database is available at the NORMAN Substance Database. For more information about the data-dependent acquisition processing see SI 2, and for confirmation with references standards, see SI 3. Relative Peak Areas (RPA) were calculated by dividing the absolute chromatographic peak areas of each analyte by the peak area of the internal standard metconazole-d6, selected for its intermediate RT, and then taking the average of these ratios.

### Exploratory Analysis

Differences between sampling days and sample preparation methods were studied using Principal Component Analysis (PCA).^27,28^ For this purpose, the peak areas of the identified compounds were extracted for each sample and evaluated using the *Statistical Analysis (one factor)* program through the MetaboAnalyst 6.0 platform. In this analysis, the three daily samples were treated as replicates of each type of sample treatment and the data were also grouped according to sample treatment type.

### Environmental Risk Assessment and Prioritization

To evaluate the persistence, the probability of aerobic and anaerobic biodegradation of organic compounds was estimated using QSAR models via *EPI Suite version 4.1* employing BIOWIN 2, 3, and 6 models. These models were utilized to classify a substance as “*Potentially Persistent (P) or very Persistent (vP)*” according to the two options indicated in Table S2. Regarding bioaccumulation and mobility, an estimation of the intrinsic physical-chemical properties, such as logarithmic octanol–water (log *K*_ow_) and organic carbon–water (log *K*_oc_) partition coefficients, was performed using the same software. Compounds with log *K*_ow_ > 4.5 were assigned as potentially bioaccumulative (B) or very bioaccumulative (vB) in aquatic biota, whereas for evaluating the mobility of compounds in soil, generic thresholds of log *K*_oc_ < 4 and log *K*_oc_ < 3 were used to assign a compound as mobile (M) or very mobile (vM), respectively (Table S2).

Substance toxicity was assessed on the basis of the predicted no-effect concentration (PNEC) of the compound in freshwater. For substances included in the EU Watch Lists, the corresponding maximum acceptable method detection or quantification limit was taken as PNEC. Meanwhile, for priority substances listed in the Directive 2013/39/EU,^19^ the PNEC corresponded to the lowest Environmental Quality Standards (EQS) in surface water. The other values were either obtained through experimental data or predicted by QSAR models from the NORMAN Ecotoxicology Database (https://www.norman-network.com/nds/ecotox/).

Compounds were then prioritized taken into account all these parameters and their abundance. To this end, percentiles (20th, 40th, 60th, and 80th) were calculated for the PNEC and the relative peak area parameters and were assigned the scores described in Table S3.

## Results and Discussion

### Identified Compounds

After peak picking, 2012, 1015, and 1749 features were annotated with SP methods 1, 2, and 3, respectively, and library matching reduced afterward these numbers to 115, 37, and 49 identifications. In total, 119 compounds were found in the effluent wastewater samples analyzed. Among them, 22 compounds were further confirmed with a confidence level of 1, “*Confirmed structure*”, after measuring the corresponding reference standard. The confirmation of these compounds was done using the retention time, exact mass, and the mSigma and MS^2^ scores obtained through the MetaboScape software. The remaining 97 compounds were tentatively identified with a level *2a*, “*Probable* structure”, according to Schymanski et al. scale.^23^ This involved the matching of the exact mass and MS^2^ fragments from the spectral library. It is worth noting that for some structural isomers, an unambiguous assignment could not be made due to their identical fragmentation patterns. This was the case for compounds such as ofloxacin/levofloxacin, and 2-aminophenol/3-hydroxy-2-methylpyridine/nicotinyl alcohol.

The identification details, along with the corresponding compound categories and PNECs, are provided in Table 1. Additionally, MS^2^ fragments matching the mass spectrometry database are available in Table S4, while the chromatographic peak areas obtained for each compound in each sample treatment are reported in Table S5 (SP method-1), Table S6 (SP method-2), and Table S7 (SP method-3).

**Table 1:** List of Identified Compounds in Effluent Samples Including Their CAS Number, Retention Time (RT), Molecular Formula, Ionization Mode, m/z, Chemical Category, Confidence Level, and Detected Method^a^

**N°**	**compound**	**CAS N°**	**RT (min)**	**molecular formula**	**ESI**	***m*/*z***	**category**	**confidence level**	detected method (*)
1	1,2,3-benzotriazole	95-14-7	5.8	C6H5N3	±	120.0561/118.0405	industrial chemicals	1	A, B, C
2	1,8-diazabicyclo [5.4.0]undec-7-ene	6674-22-2	3.6	C9H16N2	+	153.1392	industrial chemicals	2a	A, C
3	10,11-dihydro-10,11-dihydroxycarbamazepine	35079-97-1	6.7	C15H14N2O3	+	271.1083	pharmaceuticals	2a	A
4	10-hydroxycarbazepine	29331-92-8	7.3	C15H14N2O2	+	255.1134	pharmaceuticals	2a	A
5	1-naphthol	90-15-3	6.6	C10H8O	–	145.0653	pharmaceuticals/pesticides	2a	A, B
6	2,4-diaminotoluene	95-80-7	2.5	C7H10N2	+	123.0922	industrial chemicals	2a	A, C
7	2-amino-4-cresol	95-84-1	2.4	C7H9NO	+	124.0762	industrial chemicals	2a	A, B, C
8	2-amino-6-methylmercaptopurine	1198-47-6	4.2	C6H7N5S	+	182.0500	pharmaceuticals	2a	A, B
9	2-aminophenol/3-Hydroxy-2-methylpyridine/Nicotinyl alcohol	95-55-6	2.2	C6H7NO	+	110.0606	industrial chemicals	2a	A, C
10	2-methoxy-5-methylaniline	120-71-8	3.6	C8H11NO	+	138.0919	industrial chemicals	1	A, B, C
11	2-methyl-S-benzothiazole	615-22-5	11.7	C8H7NS2	+	182.0098	pesticide/industrial chemicals	2a	A, C
12	2-phenylbenzimidazole-5-sulfonic acid (Ensulizole)	27503-81-7	4.8	C13H10N2O3S	+	275.0490	industrial chemicals	2a	A
13	3,5-ditert-Butyl-4-hydroxybenzoic acid	1421-49-4	12.8	C15H22O3	+	251.1647	industrial chemicals	2a	A
14	3-[(hexanoyloxy)ethanimidoyl]-1H-pyrrole	-	8.1	C12H18N2O2	+	223.1447	other	2a	A
15	4,4′-dihydroxybiphenyl	-	9.4	C12H10O2	–	187.0759	industrial chemicals	2a	A, C
16	4-acetamidoantipyrine	83-15-8	5.3	C13H15N3O2	+	246.1243	pharmaceuticals	2a	A, B, C
17	4-formylaminoantipyrine	1672-58-8	5.2	C12H13N3O2	+	232.1086	pharmaceuticals	2a	A
18	4-methylbenzotriazole	29878-31-7	7.0	C7H7N3	±	134.0718/132.0561	industrial Chemicals	2a	A, B, C
19	5-methyl-1H-benzotriazole	136-85-6	7.1	C7H7N3	±	134.0718/132.0562	industrial Chemicals	1	A, B, C
20	6-methoxyquinoline	5263-87-6	4.1	C10H9NO	+	160.0762	industrial Chemicals	2a	A
21	6-methyl-2-pyridinemethanol	1122-71-0	3.6	C7H9NO	+	124.0762	industrial Chemicals	2a	A, B, C
22	acetaminophen	103-90-2	5.9	C8H9NO2	–	152.0711	pharmaceuticals	2a	A
23	amantadine	768-94-5	5.3	C10H17N	+	152.1439	pharmaceuticals	2a	A
24	amisulpride	71675-85-9	5.7	C17H27N3O4S	+	370.1801	pharmaceuticals	2a	A, C
25	amphetamine	300-62-9	3.6	C9H13N	+	136.1126	drugs of abuse	2a	A
26	ampyrone	83-07-8	4.1	C11H13N3O	+	204.1137	pharmaceuticals	2a	A
27	articaine	23964-58-1	6.0	C13H20N2O3S	+	285.1273	pharmaceuticals	2a	A, B, C
28	atenolol	29122-68-7	4.0	C14H22N2O3	+	267.1709	pharmaceuticals	2a	A, B, C
29	atenolol acid (metoprolol acid)	56392-14-4	5.1	C14H21NO4	+	268.1549	pharmaceuticals	2a	A, B, C
30	benzydamine	642-72-8	8.8	C19H23N3O	+	310.1919	pharmaceuticals	2a	A
31	betahistine	5638-76-6	3.9	C8H12N2	+	137.1079	pharmaceuticals	2a	B, C
32	bis(2-ethylhexyl) amine	106-20-7	10.4	C16H35N	+	242.2848	industrial Chemicals	2a	A, C
33	bisoprolol	66722-44-9	7.2	C18H31NO4	+	326.2331	pharmaceuticals	2a	A, B, C
34	bupropion	34911-55-2	7.2	C13H18ClNO	+	240.1155	pharmaceuticals	2a	A
35	caffeine	58-08-2	5.2	C8H10N4O2	+	195.0882	other	2a	A
36	caprolactam	105-60-2	4.6	C6H11NO	+	114.0919	industrial chemicals	1	A
37	carbamazepine	298-46-4	9.3	C15H12N2O	+	237.1028	pharmaceuticals	2a	A, C
38	chlorthiazide	58-94-6	4.8	C7H6ClN3O4S2	–	295.9567	pharmaceuticals	2a	A
39	citalopram	59729-33-8	8.4	C20H21FN2O	+	325.1716	pharmaceuticals	1	A, B, C
40	clarithromycin	81103-11-9	9.3	C38H69NO13	+	748.4847	pharmaceuticals	1	A
41	clindamycin	18323-44-9	7.0	C18H33ClN2O5S	+	425.1877	pharmaceuticals	2a	A, B, C
42	clopidogrel carboxylic acid	144457-28-3	6.8	C15H14ClNO2S	+	308.0512	pharmaceuticals	2a	A
43	cordycepin	73-03-0	2.3	C10H13N5O3	+	252.1097	pharmaceuticals	2a	A
44	darunavir	206361-99-1	11.3	C27H37N3O7S	+	548.2431	pharmaceuticals	2a	A
45	decanamide	2319-29-1	11.4	C10H21NO	+	172.1701	other	2a	A
46	decanophenone	6048-82-4	13.3	C16H24O	+	233.1905	other	2a	A, B, C
47	DEET	134-62-3	10.3	C12H17NO	+	192.1388	pesticides	1	A, C
48	desacetyl diltiazem	42399-40-6	7.7	C20H24N2O3S	+	373.1586	pharmaceuticals	2a	A, C
49	desmethylcitalopram	62498-67-3	8.3	C19H19FN2O	+	311.1560	pharmaceuticals	2a	A, C
50	dextromethorphan	125-71-3	7.9	C18H25NO	+	272.2014	pharmaceuticals	2a	A
51	dibutyl adipate	105-99-7	13.5	C14H26O4	+	259.1909	industrial chemicals	2a	A
52	dibutyl hydrogen phosphate	107-66-4	13.8	C8H19PO4	+	211.1099	industrial chemicals	2a	C
53	dibutyl phthalate	84-74-2	15.2	C16H22O4	+	279.1596	industrial chemicals	2a	A, C
54	diclofenac	15307-86-5	12.7	C14H11Cl2NO2	+	296.0245	pharmaceuticals	2a	A
55	diethyl phthalate	84-66-2	11.7	C12H14O4	+	223.0970	industrial chemicals	2a	A
56	diltiazem	56209-45-1	8.6	C22H26N2O4S	+	415.1692	pharmaceuticals	2a	A, C
57	diphenyl phosphate	838-85-7	6.5	C12H11O4P	–	251.0473	industrial chemicals	2a	A
58	dodecyl sulfate	151-41-7	10.0	C12H26O4S	–	267.1630	industrial chemicals	2a	A
59	dodecylbenzenesulfonic acid	121-65-3	11.1	C18H30O3S	–	327.1994	industrial chemicals	2a	A
60	doxylamine	76210-47-4	4.9	C17H22N2O	+	271.1810	pharmaceuticals	2a	A, C
61	erucamide	112-84-5	20.3	C22H43NO	+	338.3423	industrial chemicals	2a	A
62	esomeprazole	119141-88-7	6.8	C17H19N3O3S	+	346.1225	pharmaceuticals	2a	A
63	fenofibric acid	42017-89-0	12.7	C17H15ClO4	+	319.0737	pharmaceuticals	2a	A
64	fexofenadine	83799-24-0	9.3	C32H39NO4	+	502.2957	pharmaceuticals	2a	A
65	flecainide	54143-55-4	8.6	C17H20F6N2O3	+	415.1456	pharmaceuticals	1	A, B
66	florfenicol	73231-34-2	7.8	C12H14Cl2FNO4S	–	358.0083	pharmaceuticals	2a	A
67	fluconazole	86386-73-4	6.6	C13H12F2N6O	+	307.1119	pharmaceuticals	2a	A
68	flufenamic acid	530-78-9	13.6	C14H10F3NO2	±	282.0741/280.0585	pharmaceuticals	2a	A, B, C
69	gabapentin	60142-96-3	4.5	C9H17NO2	+	172.1338	pharmaceuticals	2a	A
70	galaxolidone	507442-49-1	15.3	C18H24O2	+	273.1855	other	2a	A, B, C
71	gemcitabine	95058-81-4	1.9	C9H11F2N3O4	+	264.0796	pharmaceuticals	2a	A
72	hexamethylenetetramine	100-97-0	1.6	C6H12N4	+	141.1140	pharmaceuticals	2a	B
73	hydrochlorothiazide	58-93-5	5.6	C7H8ClN3O4S2	–	297.9723	pharmaceuticals	2a	A, B
74	irbesartan	138402-11-6	9.5	C25H28N6O	+	429.2403	pharmaceuticals	2a	A, C
75	ketamine	6740-88-1	5.8	C13H16ClNO	+	238.0999	pharmaceuticals	1	A, B
76	ketoprofen	22071-15-4	11.0	C16H14O3	+	255.1021	pharmaceuticals	2a	A
77	lacosamide	175481-36-4	6.6	C13H18N2O3	+	251.1396	pharmaceuticals	2a	A
78	lamotrigine	84057-84-1	6.2	C9H7Cl2N5	+	256.0157	pharmaceuticals	2a	A, B, C
79	levamisole	14769-73-4	4.9	C11H12N2S	+	205.0800	pharmaceuticals	2a	A
80	lidocaine	137-58-6	5.7	C14H22N2O	+	235.1810	pharmaceuticals	2a	A
81	lincomycin	154-21-2	3.5	C18H34N2O6S	+	407.2216	pharmaceuticals	2a	A
82	losartan	114798-26-4	10.0	C22H23ClN6O	+	423.1700	pharmaceuticals	2a	A, C
83	melamine	108-78-1	1.7	C3H6N6	+	127.0732	industrial chemicals	1	A
84	mepivacaine	96-88-8	5.7	C15H22N2O	+	247.1810	pharmaceuticals	2a	A
85	metformin	657-24-9	1.6	C4H11N5	+	130.1093	pharmaceuticals	2a	A, B, C
86	methocarbamol	532-03-6	6.9	C11H15NO5	+	242.1029	pharmaceuticals	2a	A
87	mirtazapine	85650-52-8	6.0	C17H19N3	+	266.1657	pharmaceuticals	2a	A, B
88	m-Xylene-4-sulfonic acid (2,4-Dimethylbenzenesulfonic acid)	88-61-9	5.0	C8H10O3S	–	187.0429	other	2a	A, B
89	*N*,*N*′-diphenylguanidine (DPG)	102-06-7	6.0	C13H13N3	+	212.1188	industrial chemicals	1	A, C
90	*N*-[(S)-(+)-1-ethoxycarbonyl-3-phenylpropyl]-l-alanine	82717-96-2	7.1	C15H21NO4	+	280.1549	pharmaceuticals	2a	A
91	*N*-butylbenzenesulfonamide	3622-84-2	11.2	C10H15NO2S	+	214.0902	industrial chemicals	2a	A
92	*N*-ethylaniline	103-69-5	3.5	C8H11N	+	122.0970	industrial chemicals	2a	C
93	*N*-ethyl-*N*-methylcathinone	1157739-24-6	3.6	C12H17NO	+	192.1388	drugs of abuse	2a	A
94	noramidopyrine	519-98-2	4.1	C12H15N3O	+	218.1293	pharmaceuticals	2a	A, B
95	O-desmethyl venlafaxine	93413-62-8	5.8	C16H25NO2	+	264.1964	pharmaceuticals	1	A
96	ofloxacin/levofloxacin	100986-85-4	5.7	C18H20FN3O4	+	362.1516	pharmaceuticals	2a	A
97	omeprazole sulfone	88546-55-8	8.1	C17H19N3O4S	+	362.1175	pharmaceuticals	2a	A
98	oxcarbazepine	28721-07-5	8.4	C15H12N2O2	+	253.0977	pharmaceuticals	2a	A, C
99	perfluorobutanesulfonic acid (PFBS)	375-73-5	8.2	C4H1F9O3S1	–	300.9580	industrial chemicals	2a	A, B
100	perfluorohexanoic acid (PFHxA)	307-24-4	8.0	C6H1F11O2	–	314.9878	industrial chemicals	2a	A
101	phenazone	60-80-0	6.2	C11H12N2O	+	189.1028	pharmaceuticals	2a	A, B, C
102	pilocarpine	92-13-7	5.7	C11H16N2O2	+	209.1290	pharmaceuticals	2a	A
103	pirlimycin	79548-73-5	7.1	C17H31ClN2O5S	+	411.1721	pharmaceuticals	2a	A, B, C
104	propranolol	525-66-6	7.8	C16H21NO2	+	260.1651	pharmaceuticals	2a	A, C
105	pyrimethanil	53112-28-0	8.9	C12H13N3	+	200.1188	pesticides	2a	A
106	pyroquilon	57369-32-1	3.8	C11H11NO	+	174.0919	pesticides	2a	A
107	sitagliptin	486460-32-6	6.9	C16H15F6N5O	+	408.1259	pharmaceuticals	1	A, B, C
108	sulfamethoxazole	144930-01-8	7.7	C10H11N3O3S	+	254.0599	pharmaceuticals	1	A
109	sulpiride	15676-16-1	4.5	C15H23N3O4S	+	342.1488	pharmaceuticals	1	A, B, C
110	tapentadol	175591-23-8	6.5	C14H23NO	+	222.1858	pharmaceuticals	2a	A, B, C
111	terbutryn	886-50-0	9.6	C10H19N5S	+	242.1439	pesticides	1	A, C
112	tiamulin	55297-95-5	9.2	C28H47NO4S	+	494.3304	pharmaceuticals	2a	A, B, C
113	trazodone	19794-93-5	7.5	C19H22ClN5O	+	372.1591	pharmaceuticals	2a	A
114	tritbutyl phosphate	126-73-8	14.0	C12H27O4P	+	267.1725	industrial chemicals	1	A, B, C
115	tributylamine	102-82-9	7.8	C12H27N	+	186.2222	industrial chemicals	1	A
116	triethyl phosphate	78-40-0	7.7	C6H15O4P	+	183.0786	industrial chemicals	1	A
117	trimethoprim	738-70-5	5.4	C14H18N4O3	+	291.1457	pharmaceuticals	1	A
118	tris(2-butoxyethyl) phosphate	78-51-3	14.7	C18H39O7P	+	399.2512	industrial chemicals	1	A, B, C
119	venlafaxine	93413-69-5	7.2	C17H27NO2	+	278.2120	pharmaceuticals	1	A

a (*) **A**: Lyophilization, **B**: Direct injection, **C**: Online SPE

The identified compounds were predominantly pharmaceuticals, industrial chemicals, pesticides, and drugs of abuse. Pharmaceuticals were the most abundant (in terms of peak areas) and frequently detected category of compounds, accounting for 61.3% of detections in effluent wastewater samples (73 compounds and metabolites) (Figure 1, Figures S1 and S2). This category included antibiotics, analgesics, antihypertensive agents, and antidepressants, among others. The highest RPA was obtained for articaine, a local anesthetic used in dental interventions, whose presence, to our knowledge, has not been previously reported in environmental fate studies. In second place, lamotrigine, an antiepileptic agent commonly found in conventional wastewater effluents due to its high persistence and low biodegradation in activated sludge treatments, was also detected at elevated concentrations.^10,11,17^ O-desmethyl venlafaxine also showed high abundance, and greater than that of its parent compound, likely due to its slower biodegradation rate (the apparent elimination half-life is 5 ± 2 h for venlafaxine and 11 ± 2 h for their metabolite).^29^ The presence of O-desmethyl venlafaxine in effluent wastewater results from both human metabolism and degradation in WWTP processes.^11,30^ Other interesting identifications with RPAs greater than the 75th percentile corresponded to the antibiotics clindamycin and tiamulin. These compounds pose a potential risk to the environment due to the emergence of antibiotic resistance.^31^ In addition, at least three analgesics, including 4-acetamidoantipyrine, tapentadol, and phenazone, were found.

**Figure 1:**
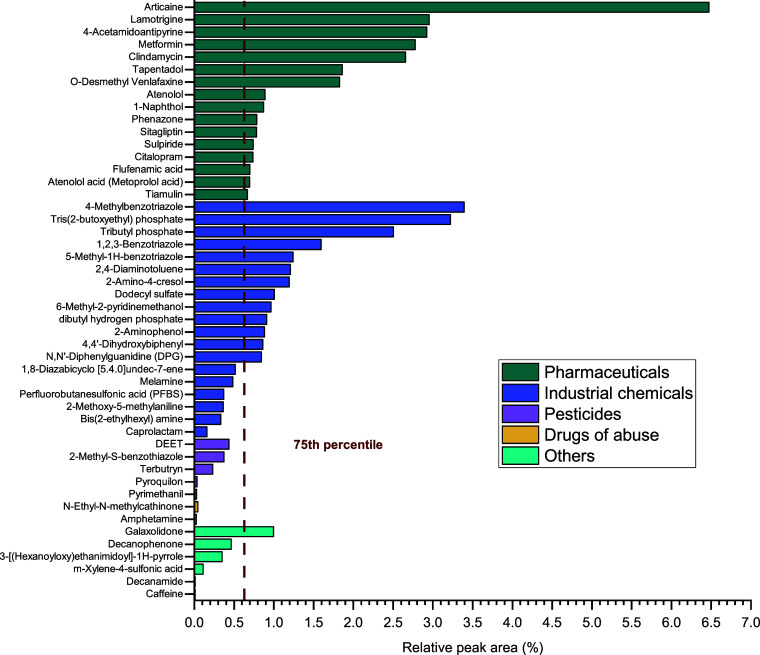
Relative peak area of the most abundant compounds by category (complete figure with all compounds in Figure S1).

Compounds used in industrial applications were the second most detected group of contaminants (a total of 33 compounds and metabolites), accounting for 27.7% of the total identifications (Figure 1 and Figures S1B and S2). This group included phthalates, flame retardants, benzotriazoles, and perfluoroalkyl compounds, among others. These substances are considered an emerging environmental concern due to their intrinsic physical-chemical properties, such as persistence and mobility.^21^ Within this category, many compounds have multiple applications, making it difficult to trace their exposure sources. For example, caprolactam is used to synthesize Nylon 6 fibers but is also employed in film coatings, synthetic leather, vehicle painting, and as a plasticizer. Among the identified industrial chemicals, benzotriazole and its metabolites 4(5)-methyl benzotriazole occupy top positions in the RPA list. The occurrence of these compounds in natural water bodies has been frequently reported in the literature, as they may persist in the environment for long periods due to their low volatility, slow biodegradation, and high polarity, making their removal in conventional WWTPs difficult.^16,21,32^

Organophosphorus flame retardants, such as tris­(2-butoxyethyl) phosphate (TBEP), tributyl phosphate and dibutyl hydrogen phosphate (DBHP or DBP), were another category detected in effluent samples with high RPAs. These nonchlorinated esters are primarily used as plasticizers, antifoaming agents, and additives.^33,34^ Toxicological studies have linked TBEP to toxic effects that pose risks to both the environment and human health.^35^ 2,4-Diaminotoluene also presented a high pollution load, possibly due to its multiple uses in hair dyes and as an intermediate for dyes, polymers, and other chemicals. It is classified as a human carcinogen by the Environmental Protection Agency (EPA).^36^ The last compound within the 75th percentile of the RPA for this category is N,N-diphenylguanidine, a guanidine-derivative used as a vulcanization accelerator in rubber products such as tires. This substance is classified as a persistent and mobile organic compound (PMOC) with accumulation potential in the aquatic environment.^21,37^

In the case of pesticides (4.2% of the total identifications, Figure S2), five compounds used as fungicides, herbicides, and insecticides were detected. Terbutryn, a priority substance regulated in surface waters under Directive 2013/39/EU^19^ was identified in water samples. Despite its regulation, these results indicate the difficulty of removing it from the aquatic environment. Since the WWTP is not located in an agricultural area, these compounds may originate from domestic sewage.

Finally, the urban origin of the wastewater also resulted in the presence of the drugs of abuse amphetamine and N-ethyl-*N*-methylcathinone (1.7% of the total identifications, Figure S2), the stimulant caffeine, and galaxolidone, a metabolite of the personal care product galaxolide.

### Exploratory Analysis of the Sample Preparation Methods

The normalized peak areas of the identified compounds were used to perform the PCA. Figure 2 shows the PCA score plot (PC1 vs PC2) of the two principal components explaining the largest amount of variance (58.2% and 28.9%, respectively). This graph shows three clusters of samples corresponding to the different sample treatments applied (SP-1, SP-2 and SP-3). Specifically, PC1 clearly separates between lyophilized water samples (SP-1) and those that are only centrifuged before injection in the (online SPE) LC-HRMS system (SP-2 and SP-3). On the other hand, PC2 differentiates between the other two sample preparation methods, SP-2 and SP-3. These aggregations indicate that the pattern of CECs, in terms of presence or abundance, exhibits differences within samples subjected to distinct SP methods. However, the amount of variation between replicates within each SP method is barely noticeable. This shows the high reproducibility of the analysis and, on the other hand, the similar contaminant load of the identified CECs in the various tested weekdays.

**Figure 2:**
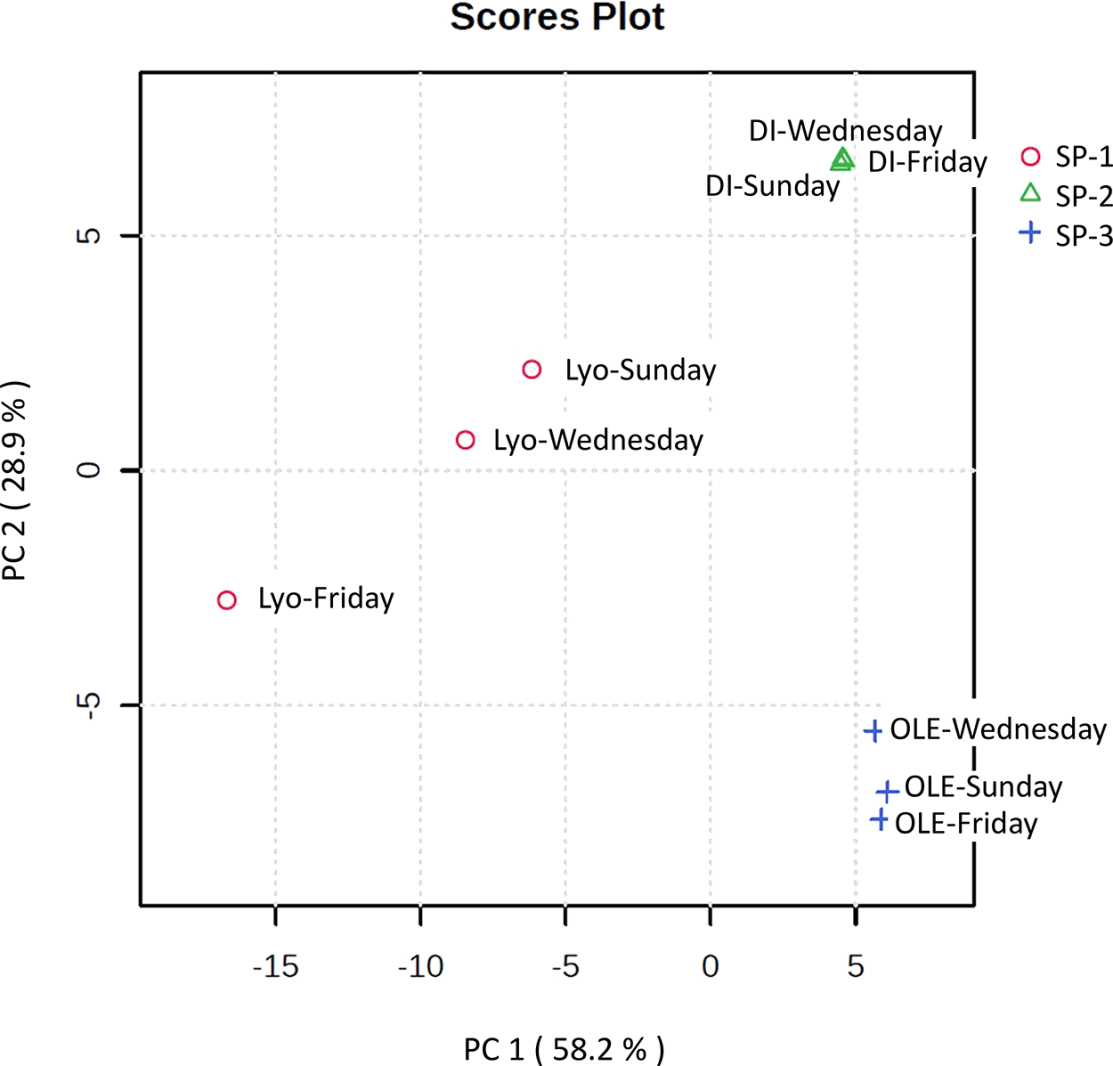
Score diagram of PC1 vs PC2, depicting three distinct clustering of samples corresponding with each sample treatment (SP-1_lyophilization “Lyo”, SP-2_direct injection “DI”, and SP-3_online SPE “OLE”).

The PCA loading plot in Figure S3 represents the variables (identified compounds) of the model. This diagram indicates that compounds with the highest values in PC1 and/or PC2 are the most important in explaining the greatest variability in the data set, regardless of whether the contribution is positive or negative.

### Compounds Identified with Each Sample Preparation Method

Sample preparation methodologies play a crucial role in the outcome of the analysis. Certain pretreatment conditions can modify the sensitivity and selectivity of the analytical method, as corroborated by the exploratory analysis in the previous section. To further understand these effects, several aspects of the three sample preparation methods are discussed and compared here.

Figure 3 shows the number of compounds within each category of use detected with each of the sample preparation methods tested, and the sum of their corresponding peak areas (the data is available in TS5-S7). Out of the total 119 compounds found in effluent wastewater, 115 were identified through sample lyophilization (SP-1), 37 with direct injection (SP-2), and 49 with online SPE (SP-3). These results demonstrate that lyophilization leads to the detection of a larger number of compounds, in spite of potential matrix effects increasing. In all cases, the predominant categories were, first, pharmaceuticals and, then, industrial chemicals. Pesticides were only detected after lyophilization and online SPE, not by direct injection, and drugs of abuse only after lyophilization, probably because of their low concentration in the treated wastewater.

**Figure 3:**
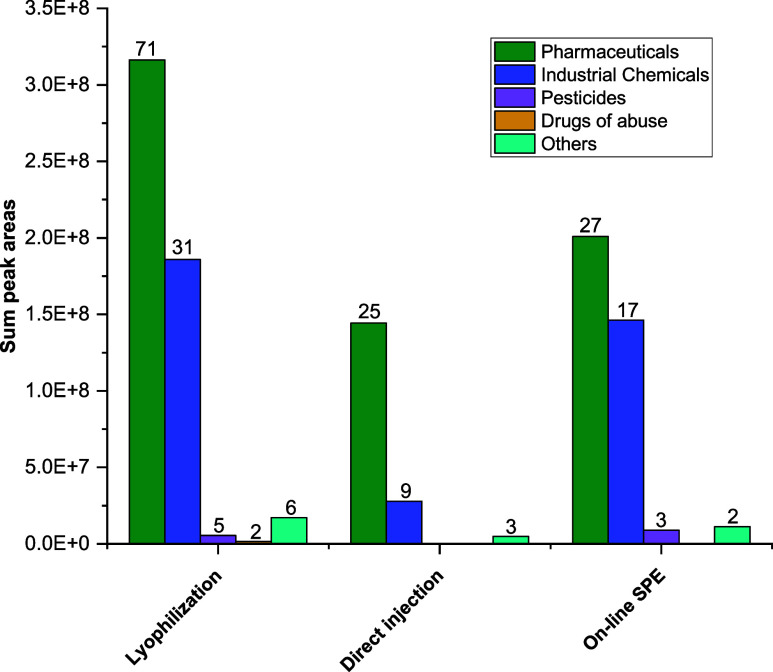
Number of CEC identified with each of the sample preparation methods tested, classified according to category of use, and sum of the corresponding peak areas.

In the case of pharmaceuticals, it is observed that, despite identifying the highest number of these compounds using lyophilization, the sum of the peak areas does not increase proportionally. The same is observed in the case of pesticides when comparing lyophilization and online SPE. This suggests that many of these compounds did not exhibit a high contaminant load in the effluent wastewater and could experiment losses during lyophilization and/or high matrix effects.

To evaluate the range of polarity, expressed as n-octanol/water partition coefficient (log *K*_ow_), organic carbon–water partition coefficient (log *K*_oc_), and Henry’s law constant of the compounds identified with each sample pretreatment method, these coefficients were estimated with QSAR models using the EPI Suite program. The obtained values are summarized in Table S8 and represented in Figure S4. Out of the three sample pretreatment methods tested, lyophilization covered the broadest range of polarity, with log *K*_ow_ values ranging from −2.34 to 8.44, followed by online SPE (log *K*_ow_ between −2.34 and 7.91), and direct injection (log *K*_ow_ between −4.15 and 5.60), which allowed the detection of more polar compounds than the other two. These polar compounds could have been lost in the lyophilization approach due to the solvents used for extract reconstitution (MeOH and EtOAc, but not H_2_O) and in the online SPE approach because of the characteristics of the polymeric sorbent used for extraction of the samples.

Regarding log *K*_oc_, values up to 6.38, corresponding to the antidiabetic agent sitagliptin, were reached with all three methods. However, lyophilization exhibited the broadest range, reaching log *K*_oc_ values as low as 0.82, corresponding to fexofenadine. Lyophilization was also the method affording the identification of the most volatile compounds.

Figure 4 shows in a Venn diagram the number of compounds identified with each sample preparation method (SPM). Twenty-six compounds, with a polarity range between log K_ow_ −2.34 and 5.60, were detected with all three methods. Twenty-three out of these 26 compounds showed high abundance in the samples, based on their chromatographic peak areas (TS5-S7), which explains their detection with all three methods, including the one that is, in principle, least sensitive (direct injection). The remaining three compounds exhibited medium to low polarity, which also facilitates their detection with all three methods. Sixty compounds were exclusively detected with the lyophilization method, one compound (the antibiotic hexamethylenetetramine, with log *K*_ow_ −4.15) with the direct injection method, and two compounds (the organophosphorus flame retardant dibutyl hydrogen phosphate and the industrial chemical N-ethylaniline, with log *K*_ow_ 2.29 and 2.16, respectively) with the online SPE method. Apart from these three compounds, the other compound missed by the lyophilization method and detected with the other two methods was the pharmaceutical betahistine, used to treat the symptoms of Ménière’s disease, with log *K*_ow_ 0.68. Twenty compounds were detected by both lyophilization and online SPE. These compounds were not highly abundant in the samples and, therefore, were only detectable due to the preconcentration of the water.

**Figure 4:**
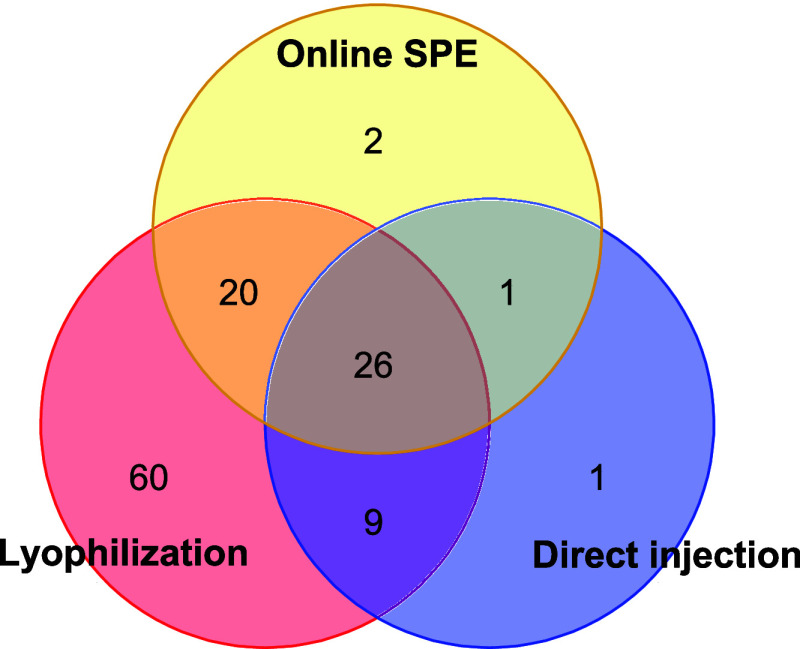
Van Venn diagrams showing the number of compounds identified with each sample pretreatment procedure and coincidences among them.

### Screening Environmental Assessment and Prioritization

The risk posed by the presence of these CECs in the environment is highlighted by the extent of emissions, their persistence, and the ease with which they can be transported across natural barriers from their point of release. Moreover, in the aquatic environment, these chemicals may present diverse toxicity and bioaccumulation potential depending on their intrinsic physical-chemical properties and the climatic conditions.

To assess the environmental fate of the compounds detected in wastewater effluents in the aquatic environment of the area under study, their persistence, bioaccumulation potential, and mobility were evaluated by classifying them as “*Potentially P or vP*”, “*Potentially B or vB in aquatic organisms*“, mobile (M), or very mobile (vM), following the criteria proposed by Neumann et at.^38^ and ECHA.^39^ This evaluation was performed considering the information compiled in Table S8. The classification of each environmental fate category and the score assigned to each of them for prioritization purposes is provided in Table S3.

Regarding persistence, up to 48 compounds were clearly classified as *Potentially P or vP*, while 23 of the remaining compounds were ambiguous and did not meet the criteria. For these, a definitive assessment of their P/vP properties should be performed based on degradation half-life testing. Concerning substances mobility, 67 and 33 compounds were categorized as vM and M, respectively. Finally, in terms of bioaccumulation, only 10 compounds, namely, bis­(2-ethylhexyl) amine, decanophenone, dibutyl phthalate, diclofenac, dodecylbenzenesulfonic acid, erucamide, flufenamic acid, galaxolidone, irbesartan, and tiamulin were considered *Potentially B or vB.*

For *PBT assessment*, the list established by ECHA in which PMT (persistent, mobile and toxic) or vPvM (very persistent and very mobile) substances are provided, was also considered. Thus, the compounds melamine, perfluorobutanesulfonic acid, and perfluorohexanoic acid, included in this list, coincided with the classification performed, while the compounds 1,2,3-benzotriazole and its derivatives, 2-methyl-S-benzothiazole, dibutyl phthalate and bis­(2-ethylhexyl) amine did not match with the persistence results obtained in the exercise performed. This shows that an estimation of persistence based on the compound structure is not sufficient since for some degradable substances, other factors must be considered. For instance, continuous delivery to the environment may render them pseudopersistent. This would be the case of the benzotriazole family.^12,40^

Prioritization of the tentatively identified CECs in the effluent wastewater samples was based on their abundance across all samples and their environmental implications, i.e., persistence, mobility, bioaccumulation and PNEC values in fresh water. For this, relative chromatographic peak areas were used as indicators of abundance since previous studies demonstrated that they provide a relatively reliable approximation to semiquantification methods (around 80%).^6^

Table S9 summarizes the individual scores attained by each compound based on these parameters. Table 2 provides a list of the top-prioritized compounds according to the total score obtained, together with their classification in terms of persistence, mobility, and bioaccumulation, and their PNECs. Twenty-five compounds, including 13 pharmaceuticals, 8 industrial chemicals, 1 personal care product, 2 pesticides, and 1 research chemical, had a total score greater than 4.5. In the top positions were the antibiotic clindamycin and the analgesic, anti-inflammatory and antipyretic flufenamic acid, both showing the highest possible total score of 6. These were followed by galaxolidone, O-desmethyl venlafaxine, and the antibiotic tiamulin, all with a total score of 5.5. It may be worth highlighting that ofloxacin and diclofenac are found to be in the top 25 due to their potential persistence, mobility and toxicity rather than their abundance.

**Table 2:** Classification of the Top-Prioritized Compounds as P (or vP), M (or vM) or B, PNEC, and Total Score for Prioritization^a^

			**conclusion of classification**					
**rank**	**compound**	**CAS N°**	**P (op1)**	**P (op2)**	**M**	**B**	**PNEC freshwater (μg/L)**	**total SCORE**
1	clindamycin	18323-44-9	Pot. P or vP	Pot. P or vP	vM	not B and not vB	0.044	6
2	flufenamic acid	530-78-9	Pot. P or vP	Pot. P or vP	M	Pot. B or vB	0.4	6
3	galaxolidone	507442-49-1	not P or vP	Pot. P or vP	not M	Pot. B or vB	0.1	5.5
4	O-desmethyl venlafaxine	93413-62-8	Pot. P or vP	Pot. P or vP	M	not B and not vB	0.006	5.5
5	tiamulin	55297-95-5	Pot. P or vP	Pot. P or vP	not M	Pot. B or vB	0.25	5.5
6	venlafaxine	93413-69-5	Pot. P or vP	Pot. P or vP	M	not B and not vB	0.006	5
7	2,4-diaminotoluene	95-80-7	Pot. P or vP	Pot. P or vP	vM	not B and not vB	12	5
8	2-amino-6-methylmercaptopurine	1198-47-6	Pot. P or vP	Pot. P or vP	vM	not B and not vB	0.49	5
9	bis(2-ethylhexyl) amine	106-20-7	not P or vP	not P or vP	not M	Pot. B or vB	0.52	5
10	decanophenone	6048-82-4	not P or vP	not P or vP	M	Pot. B or vB	0.07	5
11	diclofenac	15307-86-5	Pot. P or vP	Pot. P or vP	vM	Pot. B or vB	0.05	5
12	terbutryn	886-50-0	Pot. P or vP	Pot. P or vP	vM	not B and not vB	0.34	5
13	hexamethylenetetramine	100-97-0	Pot. P or vP	Pot. P or vP	vM	not B and not vB	11	4.5
14	1,2,3-benzotriazole	95-14-7	not P or vP	not P or vP	vM	not B and not vB	19	4.5
15	4-methylbenzotriazole	29878-31-7	not P or vP	not P or vP	M	not B and not vB	5.9	4.5
16	articaine	23964-58-1	not P or vP	Pot. P or vP	vM	not B and not vB	4.02	4.5
17	dibutyl phthalate	84-74-2	not P or vP	not P or vP	M	Pot. B or vB	10	4.5
18	lamotrigine	84057-84-1	Pot. P or vP	Pot. P or vP	M	not B and not vB	8	4.5
19	melamine	108-78-1	Pot. P or vP	Pot. P or vP	vM	not B and not vB	360	4.5
20	*N*,*N*′-diphenylguanidine (DPG)	102-06-7	not P or vP	Pot. P or vP	M	not B and not vB	1.05	4.5
21	ofloxacin/levofloxacin	100986-85-4	Pot. P or vP	Pot. P or vP	vM	not B and not vB	0.026	4.5
22	perfluorobutanesulfonic acid (PFBS)	375-73-5	Pot. P or vP	Pot. P or vP	vM	not B and not vB	372	4.5
23	phenazone	60-80-0	not P or vP	not P or vP	vM	not B and not vB	1.1	4.5
24	pirlimycin	79548-73-5	Pot. P or vP	Pot. P or vP	vM	not B and not vB	1.61	4.5
25	2-methyl-*S*-benzothiazole	615-22-5	not P or vP	not P or vP	M	not B and not vB	0.69	4.5

a **Pot.**: Potentially, **P**: Persistent, **vP**: very Persistent, **M**: Mobile, **vM**: very Mobile, **B**: Bioaccumulative, **vB**: very Bioaccumulative

## Conclusions

In this study, three different sample preparation approaches—lyophilization, direct injection, and online SPE—were evaluated for their efficiency in suspect screening detection of CECs in effluent wastewater samples via LC-HRMS analysis. Of these, the lyophilization-based method provided the best results, detecting up to 115 compounds, compared to 49 and 37 compounds detected using online SPE and direct injection, respectively. Lyophilization also offered the greatest coverage across the log *K*_ow_ range of the detected compounds: from −2.34 to 8.44, versus −2.34 to 7.91 with online SPE and −4.15 to 5.60 with direct injection. Sixty compounds were exclusively detected using the lyophilization method, one compound (hexamethylenetetramine, with log *K*_ow_ −4.15) was detected only with the direct injection method, and two compounds (dibutyl hydrogen phosphate and N-ethylaniline, with log *K*_ow_ values of 2.29 and 2.16, respectively) were exclusive to the online SPE method. The only compound missed by the lyophilization method but detected with the other two methods was the pharmaceutical betahistine, with log *K*_ow_ 0.68. The very high polarity of hexamethylenetetramine (the compound with the lowest log *K*_ow_ among all detected compounds) and the presence of matrix effects in the analysis of the other three compounds in the lyophilization method could explain these results.

These findings suggest that, for a comprehensive characterization of CECs in wastewater effluent, lyophilization would be the preferred sample preparation method among those tested. However, if the focus of the study is on more polar compounds, direct injection may be more suitable. Thus, this study provides a framework for designing optimal sample preparation strategies based on the specific objectives of the study, the compounds of interest, or their characteristics (such as polarity) in a cost-effective manner.

The results also indicate that conventional wastewater treatment processes are a source of CECs to the aquatic environment, some of which are persistent, mobile, and bioaccumulative in aquatic organisms, thus posing a potential environmental risk. These findings highlight the need for developing and implementing advanced treatment technologies to improve the removal of these pollutants. This is particularly important in the context of climate change and water scarcity, where wastewater reuse is emerging as a viable water management strategy to ensure the sustainability of water resources in various circular economy scenarios. In this regard, the application of NTS methodologies for the comprehensive characterization of pollutants in water proves to be a powerful tool. It allows for the detection of chemicals that might otherwise go unnoticed, as demonstrated in this study with the anesthetic articaine, which, to the best of the authors’ knowledge, has not been previously reported in the aquatic environment.

## Supplementary Material



## Notes

**SI 1**. LC-HRMS analysis. **SI 2**. Postacquisition data processing. **SI 3**. Confirmation procedure. **Table S1**. Isotopically labeled compounds used as internal standards for quality control. **Table S2**. Screening information, criteria and conclusion for compound persistence (P), bioaccumulation (B) and mobility (M) assessment. **Table S3**. Scores for prioritization of the identified compounds according to their persistence, bioaccumulation, mobility, toxicity, and abundance. **Table S4**. Experimental fragments matched with the mass spectrometry library of the identified compounds. **Table S5**. Chromatographic peak area obtained for each compound in sample preparation 1 (lyophilization). **Table S6**. Chromatographic peak area obtained for each compound in sample preparation 2 (direct injection). **Table S7**. Chromatographic peak area obtained for each compound in sample preparation 3 (online SPE). **Table S8**. PNEC and physical-chemical properties of the compounds identified estimated with QSAR models using EPI Suite program. **Table S9**. Individual and total scores for prioritization. **Figure S1**. (A) Relative peak area of pharmaceuticals and (B) the remaining categories. **Figure S2**. Percentage of suspect compounds identified in all samples per CEC category. **Figure S3**. PCA loading plot representing the variables (identified compounds) of the model. **Figure S4**. Range of n-octanol/water partition coefficients (log *K*_ow_), organic carbon–water partition coefficients (log *K*_oc_) and Henry’s law constant of the compounds identified with each tested sample pretreatment procedure (PDF)
