# Supplementary material for: Evaluation of Three Sample Preparation Methods for LC-HRMS Suspect Screening of Contaminants of Emerging Concern in Effluent Wastewater
Source: Anal Chem. 2025 Nov 19;97(47):25946–57. doi: 10.1021/acs.analchem.5c01659 (PMC12676514; doi:10.1021/acs.analchem.5c01659)
Supplement: Supplementary file 1 [file ac5c01659_si_001.pdf]

SUPPORTING INFORMATION

Evaluation of Three Sample Preparation Methods for LC-HRMS Suspect Screening of Contaminants of Emerging Concern in Effluent Wastewater

Dana Orlando-Véliz[1,2*], Rocío Inés Bonansea[1], Manuel Garcia-Vara[1], Varvara Nikolopoulou[3], Miren López de Alda[1*]

[1]Water, Environmental and Food Chemistry Research Unit, Dep. of Environmental Chemistry, Institute for Environmental Assessment and Water Research (IDAEA-CSIC), C/Jordi Girona 18–26, 08034 Barcelona, Spain

[2]PhD student in the Analytical and Environmental Chemistry PhD Program at the University of Barcelona, C/Martí i Franquès, 1-11, Les Corts, 08028 Barcelona, Spain

[3]Department of Environmental Health Sciences, Yale School of Public Health, Yale University, New Haven, Connecticut 06510, United States

Corresponding authors:

Miren López de Alda
Water, Environmental and Food Chemistry Research Unit (ENFOCHEM)
Institute for Environmental Assessment and Water Research (IDAEA-CSIC), Barcelona, Spain
E-mail address: mlaqam@cid.csic.es

Dana Orlando Véliz
Water, Environmental and Food Chemistry Research Unit (ENFOCHEM)
Institute for Environmental Assessment and Water Research (IDAEA-CSIC), Barcelona, Spain
E-mail address: dovqal@cid.csic.es

Number of pages: 34

Number of text information: 3

Number of tables: 9

Number of figures: 4

Table of contents

SI 1. LC-HRMS analysis 3

SI 2. Postacquisition data processing 4

SI 3. Confirmation procedure 5

Table S1. Isotopically labeled compounds used as internal standards for quality control. 6

Table S2. Screening information, criteria, and conclusion for compound persistence (P), bioaccumulation (B) and mobility (M) assessment. 7

Table S3. Scores for prioritization of the identified compounds according to their persistence, bioaccumulation, mobility, toxicity, and abundance. 8

Table S4. Experimental fragments matched with the mass spectrometry library of the identified compounds. 9

Table S5. Chromatographic peak area obtained for each compound in sample preparation 1 (lyophilization). 13

Table S6. Chromatographic peak area obtained for each compound in sample preparation 2 (direct injection). 16

Table S7. Chromatographic peak area obtained for each compound in sample preparation 3 (online SPE). 19

Table S8. PNEC and physical-chemical properties of the compounds identified estimated with QSAR models using EPI Suite program. 22

Table S9. Individual and total scores for prioritization. 27

Fig. S1. Relative peak area of A) pharmaceuticals and B) the remaining categories 31

Fig. S2. Percentage of suspect compounds identified in all samples per CEC category. 32

Fig. S3. PCA loading plot representing the variables (identified compounds) of the model. 33

Fig. S4. Range of n-octanol/water partition coefficients ($logKow$), organic carbon-water partition coefficients ($logKoc$), and Henry's law constant of the compounds identified with each tested sample pretreatment procedure. 34

SI 1. *LC-HRMS analysis*

LC-HRMS analysis was performed using an Elute UHPLC system coupled to an Impact II Q-TOF mass spectrometer (Bruker Daltonics, Billerica, MA, USA) provided with a Vacuum Insulated Probe Heated Electrospray Ionization (VIP-HESI).

Chromatographic separation of the analytes was carried out with a reversed-phase Luna Omega Polar C18 column (3µm 50 x 2.1 mm, Phenomenex, Torrance, CA, USA). The column oven was set at 30 °C, meanwhile the sample compartment temperature was set at 10 °C. A flow-rate of 0.3 mL min-1 and an injection volume of 5 µL were employed for the analysis. The mobile phase compositions in positive ionization mode consisted of (A) H2O and (B) ACN both with 0.1 % formic acid and for negative ionization mode were (A) H2O with 5 mM ammonium acetate and (B) ACN. Initially, the gradient elution program was set at 95 % A for 1 min and then increased to 97 % B in 17 min, held at 97% for 2.5 min and, finally, returned to the initial conditions with a linear gradient in 1 min and held at these conditions for 2.5 min.

Mass spectrometry analyses were performed using a VIP-HESI source in positive and negative ionization modes. Ion source conditions were as follows: capillary voltage, +2500 V in positive mode and -2500 V in negative mode; dry gas flow, 8 L min-1; dry temperature, 220 °C; nebulizer pressure, 3.0 bar; probe gas flow, 4.0 L min-1 at 300 °C.

Full-scan spectrum was recorded between mass range of 70 m/z and 1,000 m/z at a collision energy of 6.0 eV (instrument resolution of 60 000 at 1000 m/z). MS2 data were obtained in two acquisition modes The first mode, AutoMSMS, is a data dependant mode (DDA) in which the MS2 spectra of the precursor ions with the highest intensity were acquired applying a nominal collision energy (n.c.e) of 25 eV with a ramped scans range between 25 - 60 eV (100% to 240 % n.c.e.) and spectra rate of 12 Hz. The other acquisition mode, broadband Collision Induced Dissociation (bbCID), is a data independent mode (DIA) in which all ions were fragmented with a nominal collision energy of 40 eV equal to a scan range of 20 - 60 eV (50% to 150 % n.c.e.).

***SI 2**. Post-acquisition data processing*

The retrospective analyses of the samples involved comparison with the suspect list mzCloud (which contains approximately 8,000 CECs from various applications). This database, available at the NORMAN Substance Database (https://www.norman-network.com/nds/), provides detailed information on compounds such as name, SMILES ID, molecular formula and exact monoisotopic and molecular ion mass ($[M+H]^+$ and $[M-H]^-$), adducts, etc. Data-dependent acquisition was processed with the selected suspect list through MetaboScape® 2022b (Bruker Daltonics, Billerica, MA, USA), a software for compound identification from nontargeted workflows. Firstly, compounds were tentatively identified based on the exact mass (m/z) with a mass error tolerance of < 5 ppm and scoring of the difference between the measured isotopic pattern and the theoretical pattern of the ion (mSigma, narrow score: 50 and wide score: 250). The parameters applied for peak detection were: intensity threshold, 3,000 counts; minimum peak length, 8 spectra (5 spectra for recursive); feature signal, intensity. Then, the matched compounds were manually evaluated considering their MS2 spectra and compared to the fragmentation pattern available in mzCloud under similar instrumental conditions, if possible. Additionally, the peak areas of the identified compounds were obtained from full-scan data in DIA raw files using the extracted ion chromatogram function in Compass Data Analysis software version 5.3 (Bruker Daltonics, Billerica, MA, USA). The identification confidence level was assigned to the tentatively identified compounds according to Schymanski et al.[1]

[1] Schymanski, E. L.; Jeon, J.; Gulde, R.; Fenner, K.; Ruff, M.; Singer, H. P.; Hollender, J. Identifying Small Molecules via High Resolution Mass Spectrometry: Communicating Confidence. Environ. Sci. Technol. 2014, 48 (4), 2097–2098. https://doi.org/10.1021/es5002105.

***SI 3**. Confirmation procedure*

To ensure the highest level of confidence (level 1) for the identified compounds, the proposed candidates are confirmed by comparing the full MS, MS^2 spectra and experimental retention time between sample and the reference standards. To achieve this, identified compounds for which analytical standards were available in the laboratory were prepared as spiked samples at a final concentration of 1 µg/L and analysed under the same analytical conditions. The spiked samples were processed as described above using MetaboScape®. To confirm the analyte of interest with probable matching reference standards, a local spectral library of reference standards was previously created in the software. Following this, the features table of the tentative compounds was annotated using this library by matching the measured accurate mass (m/z ≤ 5ppm), retention time (RT ≤ 0.5 min), isotopic fit pattern (mSigma: ≤50 narrow and ≤ 100 wide), and fragmentation score (MS2 score ≥ 80%). The mSigma score describes the isotopic fit of the ion, while the MS2 score corresponds to a value associated with the matched fragmentation pattern.

Table S1. *Isotopically labeled compounds used as internal standards for quality control.*

Compounds*	Molecular formula	***m/z*** **expected**	***m/z*** **experimental**	**RT (min) online**	**RT (min) off-line**
Fluconazole-d4	$C_{13}H_8D_4F_2N_6O$	311.1370	311.1339	6.5	6.5
Imidacloprid-d4	$C_9H_6D_4ClN_5O_2$	260.0852	260.0823	7	7
Venlafaxine-d6	$C_{17}H_{21}D_6NO_2$	284.2496	284.2464	7.2	7.3
Sulfamethoxazole-d4	$C_{10}H_7D_4N_3O_3S$	258.0850	258.0819	7.7	7.6
Dichlorvos-d6	$C_4HD_6Cl_2O_4P$	226.9913	226.9882	8.9	9
Diuron-d6	$C_9H_4D_6Cl_2N_2O$	239.0625	239.0593	10.3	10.5
Diclofenac-d4	$C_{14}H_7D_4Cl_2NO_2$	300.0496	300.0464	12.5	12.6
Metconazole-d6	$C_{17}H_{16}D_6ClN_3O$	326.1906	326.1871	12.8	13

*Standards were purchased from either Merck (Darmstadt, Germany) or Toronto Research Chemicals (Toronto, ON, Canada).

***Table S2**. Screening information, criteria, and conclusion for compound persistence (P), bioaccumulation (B) and mobility (M) assessment.*

Parameter	Criteria	Conclusion
Persistence		
Option 1	Biowin 2 (non-linear model prediction) < 0.50<br>Biowin 3 (ultimate biodegradation time) < 2.75	Potentially persistent (P) or very persistent (vP)
Option 2	Biowin 3 (ultimate biodegradation time) < 2.75<br>Biowin 6 (MITI non-linear model prediction) < 0.50	Potentially P or vP
Bioaccumulation		
	$\log K_{ow} \leq 4.5$	Not bioaccumulable (B) and very bioccumulable (vB) in aquatic organisms
	$\log K_{ow} > 4.5$	Potentially B and vB in aquatic organism
Mobility		
	$\log K_{oc} \leq 4$	Mobile (M)
	$\log K_{oc} < 3$	Very mobile (vM)
	$\log K_{oc} > 4$	Not M or vM

Table S3. *Scores for prioritization of the identified compounds according to their persistence, bioaccumulation, mobility, toxicity, and abundance.*

Factor	Category	Score
Persistence		
	Potentially P or vP	1
	Unclear P or vP (Contradictory results coming from Biowin models (Options 1 and 2 in TS-1)	0.5
	Not P or vP	0
Bioaccumulation		
	Potentially B and vB in aquatic organisms	1
	Not B or vB in aquatic organisms	0
Mobility		
	Very mobile (vM)	1
	Mobile (M)	0.5
	Not M or vM	0
PNEC		
	<20th percentile of all compounds PNEC	2
	20th – 40th percentile of all compounds PNEC	1.5
	40th – 60th percentile of all compounds PNEC	1
	60th – 80th percentile of all compounds PNEC	0.5
	>80th percentile of all compounds PNEC	0
Relative peak area		
	>80th percentile of the total peak area of compounds	2
	80th – 60th percentile of the peak total area of compounds	1.5
	60th – 40th percentile of the peak total area of compounds	1
	40th – 20th percentile of the peak total area of compounds	0.5
	<20th percentile of the total peak area of compounds	0

An extra score of 1 was considered for the compounds already categorized as PMT/PBT/PM/vPvM by UBA (German Environment Agency) (https://echa.europa.eu/pbt)

***Table S4**. Experimental fragments matched with the mass spectrometry library of the identified compounds.*

№	Compound	MS^2 Score	mSigma score	Fragments (m/z)
1	1,2,3-Benzotriazole	999	6.52	65.0429/92.0492/120.0551
2	1,8-Diazabicyclo [5.4.0]undec-7-ene	-	-	96.0807/125.107/153.1381
3	10,11-Dihydro-10,11-dihydroxycarbamazepine	-	-	167.0720/180.0794/181.0839/182.0954/ 192.0859/208.0740/210.0901
4	10-Hydroxycarbazepine	-	-	116.0498/152.0625/165.0685/167.0733/179.0727/ 192.0804/193.0874/194.0960
5	1-Naphthol	-	-	115.0551/125.0409
6	2,4-Diaminotoluene	-	-	95.0491/105.0481/106.0651/108.0665/
7	2-Amino-4-cresol	-	-	79.0556/95.0489/106.0651/107.0490/109.0522/ 124.0753
8	2-Amino-6-methylmercaptopurine	-	-	80.0239/109.0505/134.0454/165.0223/167.0255
9	2-Aminophenol/3-Hydroxy-2-methylpyridine/Nicotinyl alcohol	-	-	65.0389/82.0656/92.0500<br>67.0420/80.0501/82.0656/92.0500<br>65.0389/79.0413/80.0501/82.0656/92.0500/93.0578
10	2-Methoxy-5-methylaniline	821	71.28	79.0054/79.06501/105.0435/106.0631/108.0447/ 120.0810/122.0598/123.0671
11	2-Methyl-S-benzothiazole	-	-	68.9792/109.0106/135.0134/149.0234/166.9853
12	2-Phenylbenzimidazole-5-sulfonic acid (Ensulizole)	-	-	91.0406/104.0485/166.0642/167.0708/182.0858/ 192.0682/193.0756/194.0814/209.0755/226.0714/ 275.0473
13	3,5-di-tert-Butyl-4-hydroxybenzoic acid	-	-	139.0396/177.0905/195.1019/196.1051/251.1646
14	3-[(Hexanoyloxy)ethanimidoyl]-1H-pyrrole	-	-	67.0417/80.0495/107.0603/125.0684
15	4,4'-Dihydroxybiphenyl	-	-	92.0263/93.0373/117.0346/118.0391/128.0630/129.0704/ 130.0460/156.0576/157.0651/167.0496
16	4-Acetamidoantipyrine (N-Acetylaminoantipyrine)	-	-	94.0648/104.0491/111.0549/145.0750/159.0909/187.0858/ 204.1124/213.0889/226.0968/228.1125/
17	4-Formylaminoantipyrine	-	-	77.0393/83.0593/85.0745/94.0639/104.0481/ 146.0585/159.0908/173.0692/187.0874/204.1121/ 214.0962/232.1061
18	4-Methylbenzotriazole	-	-	95.0490/106.0649/119.0474
19	5-Methyl-1H-benzotriazole	904	6.34	77.0385/79.0544/89.0387/91.0415/95.0492/104.0497/ 105.0449/106.0651/134.0715
20	6-Methoxyquinoline	-	-	89.0381/117.0565/132.0802
21	6-Methyl-2-pyridinemethanol	-	-	79.0538/106.0649/124.0751
22	Acetaminophen (Paracetamol)	-	-	107.0377/108.0446
23	Amantadine	-	-	65.0352/77.0369/78.0398/79.0528/81.0681/91.0536/ 93.0689/107.0834/135.1160
24	Amisulpride	-	-	112.1116/155.1171/196.0059/242.0476/259.0748/313.1231/ 353.1525
25	Amphetamine	-	-	65.0397/79.0542/91.0546/92.0580/120.0802

№	Compound	MS^2 Score	mSigma score	Fragments (m/z)
26	Ampyrone	-	-	68.0494/83.0604/85.0722/94.0651/104.0491/111.0560/118.0648/130.0649/145.0758/146.0598/159.0911/173.0708/187.0866
27	Articaine	-	-	86.0964/114.0917/140.0162/168.0110/172.0432
28	Atenolol	-	-	74.0600/98.0965/116.1069/145.0644/162.0910/180.1012/190.0861/204.1392/208.0967/225.1237/232.1354/250.1446
29	Atenolol acid (Metoprolol acid)	-	-	98.0962/116.1067/145.0641/165.0541/191.0697/226.1070/250.1432
30	Benzydamine	-	-	86.0967/107.0494/174.0784/265.1343
31	Betahistine	-	-	67.0546/78.0345/94.0660/106.0662/110.0613
32	Bis(2-ethylhexyl) amine	-	-	130.1589/242.2842
33	Bisoprolol	-	-	72.0805/74.0625/75.0438/79.0541/84.0808/91.0539/95.0488/98.0962/107.0488/116.1065/133.0643/147.0799/149.0951/162.0907/163.0748/180.1013/204.1375
34	Bupropion	-	-	103.0539/130.0648/131.0727/139.0306/166.0417/167.0251/184.0534/185.0558
35	Caffeine	-	-	110.0717/138.0668/195.0895
36	Caprolactam	825	2.14	69.0675/72.0790/79.0541/86.0964/96.0802/105.0445
37	Carbamazepine	-	-	167.0851/179.0828/192.0806/194.0964
38	Chlorthiazide	-	-	229.9561/214.9656/213.9602/182.9854/120.0071/118.0142
39	Citalopram	982	10.71	109.0449/116.0494/166.0652/221.0636/224.0869/234.0715/262.1027/280.1127/307.1607
40	Clarithromycin	788	36.54	83.0491/98.0965/113.0597/116.1070/158.1177
41	Clindamycin	-	-	126.1276/172.1331/299.1969/335.1729/341.2065/377.1890/389.2115/407.1758
42	Clopidogrel carboxylic acid	-	-	125.0156/140.0270/141.0111/152.0280/169.0051/170.0359/198.0317
43	Cordycepin	-	-	92.0247/109.0516/119.0356/136.0624/137.0404
44	Darunavir	-	-	69.0340/113.0606/120.0828/156.0122/202.1575/241.1038/245.1662/392.2028
45	Decanamide	-	-	74.0615/88.0765/102.0925/116.1050/172.1698/173.1744
46	Decanophenone	-	-	79.0542/91.0543/95.0856/107.0857/117.0696/131.0852/145.1010/149.0957/163.1125/173.1323
47	DEET	873	12.31	72.0475/100.0751/119.0485/192.1385
48	Desacetyl diltiazem	-	-	72.0808/150.0372/178.0320/223.0896/328.1002
49	desmethylcitalopram	-	-	89.0385/109.0445/116.0491/123.0229/166.0646/221.0627/234.0708/262.1020/293.1449
50	Dextromethorphan	-	-	69.0685/91.0529/121.0655/135.0812/171.0815/213.1259/215.1423/272.2007
51	Dibutyl adipate	-	-	83.0495/101.0594/111.0448/129.0552/147.0651
52	Dibutyl hydrogen phosphate	-	-	80.9736/98.9844/116.9947/155.0476
53	Dibutyl phthalate	-	-	149.0217/150.0251/151.0271/205.0841/206.0871/279.1567
54	Diclofenac	-	-	151.0560/152.0601/169.0652/177.0566/178.0640/179.0705/180.0790/214.0409/215.0480
55	Diethyl phthalate	-	-	65.0387/121.0283/149.0232/150.0266/167.0339/177.0546/178.0579
56	Diltiazem	-	-	72.0809/150.0371/178.0320/191.0399/310.0900/370.1111
57	Diphenyl phosphate	-	-	65.0404/75.0237/78.9610/93.0343/249.0312
58	Dodecyl sulfate	-	-	79.9573/96.9601/265.1479

№	Compound	MS^2 Score	mSigma score	Fragments (m/z)
59	Dodecylbenzenesulfonic acid	-	-	79.9552/170.0040/183.0121/197.0277/261.2213
60	Doxylamine	-	-	72.0808/90.0918/139.0536/167.0726/182.0962/226.1224
61	Erucamide	-	-	111.1156/114.0902/121.1006/128.1054/135.1165/142.1216/149.1330/153.1281/156.1382/177.1661/198.1857/303.3034/321.3145
62	Esomeprazole	-	-	106.0646/120.0814/136.0761/149.0707/150.0920/151.0999/168.1019/180.0487/198.0590/328.1124
63	Fenofibric acid	-	-	110.9997/121.0276/138.9997/233.0356
64	Fexofenadine	-	-	91.0548/105.0697/117.0550/129.0702/131.0860/143.0878/171.1182/177.0920/189.1234/466.2741/484.2865
65	Flecainide	954	10.93	81.0696/98.0961/209.0215/232.0964/301.0290/315.1071/398.1179
66	Florfenicol	-	-	78.9865/82.9487/119.0503/121.0288/168.9985/185.0277/209.0267
67	Fluconazole	-	-	70.0402/71.0392/82.0399/83.0491/84.0544/121.0447/127.0351/139.0358/141.0511/151.0356/169.0458/170.0496/200.0633/220.0682/221.0715/238.0785/239.0821/307.1133
68	Flufenamic acid	-	-	166.0656/176.0500/196.0561/216.0622/227.0379/234.0529/236.0688
69	Gabapentin	-	-	67.0538/91.0539/93.0695/95.0853/109.1008/119.0846/137.0956/154.1216
70	Galaxolidone	-	-	157.1008/175.1114/185.0959/203.1065/227.1491/240.1505/255.1740
71	Gemcitabine	-	-	69.0453/79.0379/87.0249/95.0244/107.0302/112.0510/264.0796
72	Hexamethylenetetramine	-	-	71.0560/83.0601/85.0760/98.0713/112.0870
73	Hydrochlorothiazide	-	-	196.0190/204.9837/214.9668/231.9942/268.9457/269.9405
74	Irbesartan	-	-	180.0800/195.1486/207.0911/318.1598/386.2225/401.2308
75	Ketamine	974	14.41	125.0140/141.0092/151.0294/163.0292/165.0093/179.0609/189.0438/207.0552/220.0885
76	Ketoprofen	-	-	77.0377/95.0480/103.0541/105.0326/121.1008/131.0547/177.0528/194.0718/209.0948
77	Lacosamide	-	-	74.0612/91.0531/108.0812/116.0692/144.0795
78	Lamotrigine	-	-	220.0379/151.0178/158.9756/166.0285/193.0396/210.9819/229.0038
79	Levamisole	-	-	88.0218/129.0689/145.0753/178.0678/188.0527
80	Lidocaine	-	-	86.0971/87.1005
81	Lincomycin	-	-	82.0660/126.1284/172.1344/359.1284/389.2161/407.2212
82	Losartan	-	-	80.0492/142.0286/171.0680/180.0802/207.0913/235.0975/258.1025/324.1490/362.1415
83	Melamine	882	12.66	68.0243/85.0511/86.0356/110.0463
84	Mepivacaine	-	-	70.0650/98.0964
85	Metformin	-	-	85.0500/88.0871/113.0823
86	Methocarbamol	-	-	75.0452/79.0547/95.0501/105.0695/118.0507/125.0601/135.0808/151.0755/163.0755
87	Mirtazapine	-	-	85.0767/144.0801/180.0486/195.0910/209.1069/223.1224/264.1501
88	m-Xylene-4-sulfonic acid (2,4-Dimethylbenzenesulfonic acid)	-	-	79.9575/106.0422/121.0624/121.0652/185.0271
89	N,N'-Diphenylguanidine (DPG)	949	6.67	77.0389/92.0530/94.0654/95.0495/105.0459/119.0610/195.0930/212.1197
90	N-[(S)-(+)-1-Ethoxycarbonyl-3-phenylpropyl]-L-alanine	-	-	91.0539/117.0978/130.0887/134.0898/160.1121/206.1179/234.1473
91	N-Butylbenzenesulfonamide	-	-	77.0376/81.0331/95.0481/105.0453/106.0428/141.0003/158.0278

Nº	Compound	MS^2 Score	mSigma score	Fragments (m/z)
92	N-Ethylaniline	-	-	66.0463/77.0392/92.0499/93.0583/94.0659/95.0496/105.0449/ 120.0809
93	N-Ethyl-N-methylcathinone	-	-	105.0707/133.0655/146.0951/192.1393
94	Noramidopyrine	-	-	70.0648/84.0688/97.0760/104.0486/125.0704/146.0585/159.0910/ 187.0848
95	O-Desmethyl Venlafaxine	942	9.04	69.0699/81.0690/107.0479/121.0634/133.0635/145.0638/ 152.1415/164.1042/173.0960/201.1263/246.1836/264.1926
96	Ofloxacin/Levofloxacin	-	-	233.0699/247.0861/261.1027/262.1021/316.1419/318.1588/ 319.1610/344.1312
97	Omeprazole sulfone	-	-	120.0808/136.0755/149.0719/150.0915/151.1000/166.0858/ 168.1024/195.0220/214.0537/244.0885/298.1554
98	Oxcarbazepine	-	-	104.0509/132.0432/165.0690/180.0800/182.0955/208.0751/ 210.0907/236.0699/237.0732
99	Perfluorobutanesulfonic acid (PFBS)	-	-	79.9574/82.9616/98.9559/168.9887/218.9890/298.9425
100	Perfluorohexanoic acid (PFHxA)	-	-	268.9817/118.9933
101	Phenazone	-	-	104.0492/120.0801/131.0723/144.0801/146.0827/161.1068/ 172.0747/174.0780
102	Pilocarpine	-	-	68.0496/80.0509/82.0663/94.0647/106.0669/108.0810/ 109.0734/112.1141/117.0554/121.0771/122.0836/ 123.0832/135.0921/137.1077/149.1081/161.1082/ 163.1238/191.1190/209.1296
103	Pirlimycin	-	-	112.1116/146.0368/224.0678/252.0629/272.0713/363.1676
104	Propranolol	-	-	86.0962/98.0970/116.1072/132.1010/157.0648/183.0799/218.1160
105	Pyrimethanil	-	-	67.0422/80.0516/82.0653/83.0617/95.0494/107.0617/ 118.0641/119.0613/129.0694/143.0618/156.0790/ 168.0674/181.0773/183.0919/200.1192
106	Pyroquilon	-	-	79.0536/91.0531/103.0525/115.0537/117.0570/132.0876/ 144.0807/146.0959/174.0911
107	Sitagliptin	962	10.14	154.0456/174.0519/193.0690/219.0487/235.0797/391.0985
108	Sulfamethoxazole	782	33.31	65.0390/68.0507/80.0494/92.0495/93.0572/94.0641/99.0555/107.0599/ 108.0447/110.0603/120.0557/146.0717/147.0794/148.0860/156.0120/ 157.0158/160.0872/173.0597/188.0829/190.0974/254.0616
109	Sulpiride	967	8.71	98.0962/112.1116/199.0052/214.0162/231.0435/297.0896/
110	Tapentadol	-	-	83.0856/107.0488/121.0644/135.0799
111	Terbutryn	894	7.62	71.0599/91.0320/158.0487/186.0802
112	Tiamulin	-	-	74.0968/119.0164/163.1121/192.1057/267.2113/285.221
113	Trazodone	-	-	78.0323/96.0448/121.0693/133.0747/148.0504/ 154.0453/176.0818/372.1576
114	Tributyl phosphate	1000	6.7	80.9755/98.9831/116.9935/155.0428
115	Tributylamine	983	9.94	74.0961/86.0961/130.1583/186.2211
116	Triethyl phosphate	999	21.07	80.9734/98.9839/116.9943/127.0145
117	Trimethoprim	964	4.67	123.0664/230.1162/245.1028/258.1107/261.0983/275.1142/291.1450
118	Tris(2-butoxyethyl) phosphate	901	20.78	83.0853/98.9842/101.0961/124.9948/143.0098/199.0725
119	Venlafaxine	935	6.93	79.0540/91.0541/121.0643/147.0799/159.0801/173.0956/ 215.1426/260.2001

MS^2 Score: value associated with the matched fragmentation pattern
mSigma Score: value related to isotopic fit pattern

***Table S5**. Chromatographic peak area obtained for each compound in sample preparation 1 (lyophilization).*

№	Compound	AREA				
		ST-1 S120122	ST-1 S140122	ST-1 S160122	ST-1 average	ST-1 sum
1	1,2,3-Benzotriazole	9,522,490	3,805,065	6,884,588	6,737,381	20,212,142
2	1,8-Diazabicyclo [5.4.0]undec-7-ene	422,868	1,581,969	648,846	884,561	2,653,683
3	10,11-Dihydro-10,11-dihydroxycarbamazepine	174,553	217,221	301,906	231,227	693,681
4	10-Hydroxycarbazepine	214,275	154,152	188,984	185,804	557,411
5	1-Naphthol	2,246,386	3,371,338	5,753,745	3,790,490	11,371,469
6	2,4-Diaminotoluene	3,736,853	2,838,505	193,212	2,256,190	6,768,569
7	2-Amino-4-cresol	2,268,865	1,626,086	1,023,847	1,639,599	4,918,798
8	2-Amino-6-methylmercaptopurine	878,489	717,382	936,858	844,243	2,532,729
9	2-Aminophenol/3-Hydroxy-2-methylpyridine/Nicotinyl alcohol	3,627,746	1,449,883	446,930	1,841,520	5,524,559
10	2-Methoxy-5-methylaniline	2,414,216	1,966,485	223,743	1,534,815	4,604,444
11	2-Methyl-S-benzothiazole	77,108	86,510	130,455	98,024	294,073
12	2-Phenylbenzimidazole-5-sulfonic acid (Ensulizole)	159,516	126,073	137,498	141,029	423,088
13	3,5-di-tert-Butyl-4-hydroxybenzoic acid	69,953	78,134	112,200	86,763	260,288
14	3-[(Hexanoyloxy)ethanimidoyl]-1H-pyrrole	3,626,873	1,783,439	3,025,326	2,811,880	8,435,639
15	4,4'-Dihydroxybiphenyl	3,338,438	88,455	88,517	1,171,804	3,515,411
16	4-Acetamidoantipyrine	8,821,408	7,457,291	7,904,320	8,061,006	24,183,019
17	4-Formylaminoantipyrine	1,395,688	1,375,916	1,344,010	1,371,871	4,115,614
18	4-Methylbenzotriazole	12,464,875	6,196,125	11,406,068	10,022,356	30,067,067
19	5-Methyl-1H-benzotriazole	11,075,017	1,774,158	3,787,642	5,545,606	16,636,818
20	6-Methoxyquinoline	430,346	305,195	-	245,180	735,541
21	6-Methyl-2-pyridinemethanol	1,348,456	1,173,674	-	840,710	2,522,130
22	Acetaminophen	404,031	357,129	407,308	389,489	1,168,468
23	Amantadine	139,221	116,668	122,891	126,260	378,780
24	Amisulpride	1,740,550	1,285,069	1,264,224	1,429,948	4,289,844
25	Amphetamine	276,474	119,982	169,724	188,727	566,180
26	Ampyrone	184,503	3,048,182	525,690	1,252,792	3,758,375
27	Articaine	12,363,328	111,808	19,074,511	10,516,549	31,549,648
28	Atenolol	1,554,787	1,600,831	96,310	1,083,976	3,251,928
29	Atenolol acid (Metoprolol acid)	3,591,851	2,697,151	2,789,676	3,026,226	9,078,677
30	Benzydamine	319,678	289,336	273,724	294,246	882,738
31	Betahistine	-	-	-	-	-
32	Bis(2-ethylhexyl) amine	689,074	797,121	579,150	688,448	2,065,344
33	Bisoprolol	1,380,521	1,000,784	5,692,950	2,691,418	8,074,255
34	Bupropion	134,041	99,433	187,639	140,371	421,113
35	Caffeine	59,701	59,853	76,303	65,286	195,858
36	Caprolactam	2,015,057	845,599	900,722	1,253,792	3,761,377
37	Carbamazepine	311,061	223,169	254,847	263,026	789,077
38	Chlorthiazide	12,304	10,663	13,208	12,058	36,175
39	Citalopram	2,103,118	1,534,197	1,603,282	1,746,866	5,240,597

№	Compound	AREA				
		ST-1 S120122	ST-1 S140122	ST-1 S160122	ST-1 average	ST-1 sum
40	Clarithromycin	23,304	20,966	24,750	23,006	69,019
41	Clindamycin	1,864,348	5,446,610	12,616,449	6,642,469	19,927,407
42	Clopidogrel carboxylic acid	103,102	80,162	81,582	88,282	264,846
43	Cordycepin	-	38,899	31,140	23,346	70,039
44	Darunavir	66,259	59,888	76,868	67,672	203,015
45	Decanamide	94,196	62,778	87,319	81,431	244,293
46	Decanophenone	1,208,619	2,909,659	161,061	1,426,446	4,279,338
47	DEET	1,007,342	886,982	798,608	897,644	2,692,932
48	Desacetyl diltiazem	189,963	138,655	136,923	155,180	465,541
49	Desmethylcitalopram	663,630	445,790	444,788	518,069	1,554,208
50	Dextromethorphan	119,972	80,587	92,678	97,746	293,237
51	Dibutyl adipate	2,996	10,259	6,205	6,487	19,460
52	Dibutyl hydrogen phosphate	-	-	-	-	-
53	Dibutyl phthalate	190,243	518,577	364,751	357,857	1,073,570
54	Diclofenac	42,301	36,921	49,141	42,788	128,363
55	Diethyl phthalate	1,161,480	945,936	1,189,070	1,098,829	3,296,487
56	Diltiazem	854,453	716,379	775,461	782,098	2,346,293
57	Diphenyl phosphate	102,977	190,945	143,019	145,647	436,941
58	Dodecyl sulfate	4,376,440	9,199,673	7,175,050	6,917,054	20,751,163
59	Dodecylbenzenesulfonic acid	-	173,654	76,170	83,275	249,824
60	Doxylamine	230,194	202,430	266,669	1,640,354	699,294
61	Erucamide	26,957	-	2,037	9,665	28,994
62	Esomeprazole	14,424	128,969	72,172	71,855	215,565
63	Fenofibric acid	34,972	29,043	40,215	34,743	104,229
64	Fexofenadine	70,547	64,389	66,370	67,102	201,306
65	Flecainide	2,912,645	2,372,843	2,442,272	2,575,920	7,727,761
66	Florfenicol	509,993	608,643	543,174	553,937	1,661,810
67	Fluconazole	95,948	62,172	87,110	81,743	245,230
68	Flufenamic acid	1,300,740	2,511,636	2,839,027	2,217,134	6,651,403
69	Gabapentin	966,077	760,345	873,234	866,552	2,599,656
70	Galaxolidone	423,193	209,196	859,440	497,276	1,491,829
71	Gemcitabine	237,180	217,591	196,357	217,043	651,128
72	Hexamethylenetetramine	-	-	-	-	-
73	Hydrochlorothiazide	1,926,073	1,114,357	1,048,002	1,362,811	4,088,432
74	Irbesartan	29,648	504,772	906,424	480,282	1,440,845
75	Ketamine	355,415	266,679	319,295	313,796	941,389
76	Ketoprofen	147,185	120,727	170,028	145,980	437,940
77	Lacosamide	254,804	325,654	258,235	279,564	838,692
78	Lamotrigine	8,816,563	8,880,980	8,752,196	8,816,580	26,449,740
79	Levamisole	145,208	86,530	129,804	120,514	361,542
80	Lidocaine	1,243,271	2,320,722	1,363,142	1,642,378	4,927,134
81	Lincomycin	280,134	260,848	220,693	253,891	761,674
82	Losartan	717,178	671,817	938,362	775,786	2,327,357
83	Melamine	3,720,221	4,480,165	1,478,016	3,226,134	9,678,401
84	Mepivacaine	2,322,018	1,163,202	2,793,513	2,092,911	6,278,733

№	Compound	AREA				
		ST-1 S120122	ST-1 S140122	ST-1 S160122	ST-1 average	ST-1 sum
85	Metformin	1,422,270	1,512,005	2,817,190	1,917,155	5,751,465
86	Methocarbamol	146,569	121,314	122,512	130,132	390,395
87	Mirtazapine	439,138	330,183	374,878	381,400	1,144,199
88	m-Xylene-4-sulfonic acid (2,4-Dimethylbenzenesulfonic acid)	760,357	616,531	1,101,745	826,211	2,478,633
89	N,N'-Diphenylguanidine (DPG)	3,202,565	2,154,308	2,114,283	2,490,385	7,471,156
90	N-[(S)-(+)-1-Ethoxycarbonyl-3-phenylpropyl]-L-alanine	194,245	164,188	221,458	193,297	579,891
91	N-Butylbenzenesulfonamide	12,276	6,621	10,683	9,860	29,580
92	N-Ethylaniline	-	-	-	-	-
93	N-Ethyl-N-methylcathinone	391,728	367,296	212,224	323,749	971,248
94	Noramidopyrine	23,725	4,073,776	311,733	1,469,745	4,409,234
95	O-Desmethyl venlafaxine	14,126,618	13,366,556	12,827,437	13,440,204	40,320,612
96	Ofloxacin / Levofloxacin	221,238	245,094	248,254	238,195	714,585
97	Omeprazole sulfone	94,789	104,865	-	66,552	199,655
98	Oxcarbazepine	235,805	113,431	227,566	192,267	576,802
99	Perfluorobutanesulfonic acid (PFBS)	2,726,727	2,467,934	2,542,663	2,579,108	7,737,324
100	Perfluorohexanoic acid (PFHxA)	91,513	103,183	92,886	95,861	287,582
101	Phenazone	3,101,391	1,412,924	2,236,327	2,250,214	6,750,642
102	Pilocarpine	238,030	263,273	278,829	260,044	780,132
103	Pirlimycin	218,110	195,762	1,100,285	504,719	1,514,156
104	Propranolol	134,659	87,898	103,775	108,777	326,332
105	Pyrimethanil	220,297	181,997	228,900	210,398	631,193
106	Pyroquilon	267,263	235,480	236,566	246,436	739,309
107	Sitagliptin	2,250,789	1,806,170	1,883,311	1,980,090	5,940,269
108	Sulfamethoxazole	82,635	61,848	49,131	64,538	193,613
109	Sulpiride	2,255,743	2,023,616	2,022,839	2,100,732	6,302,197
110	Tapentadol	5,420,557	4,580,205	4,580,074	4,860,279	14,580,836
111	Terbutryn	446,632	356,097	465,211	422,647	1,267,941
112	Tiamulin	291,719	2,249,851	2,103,945	1,548,505	4,645,515
113	Trazodone	122,443	88,779	88,408	99,877	299,631
114	Tributyl phosphate	1,758,481	4,998,604	3,081,112	3,279,399	9,838,198
115	Tributylamine	686,248	545,917	1,542,877	925,014	2,775,041
116	Triethyl phosphate	212,201	140,593	255,451	202,748	608,245
117	Trimethoprim	256,449	196,204	185,890	212,848	638,543
118	Tris(2-butoxyethyl) phosphate	5,087,698	6,086,144	5,822,496	5,665,446	16,996,339
119	Venlafaxine	5,373,393	4,124,836	3,578,544	4,358,924	13,076,772
	Metconazole-d6	2,996,847	1,670,377	3,446,364	2,704,529	8,113,588

Value “-” means compound no detected

Table S6. *Chromatographic peak area obtained for each compound in sample preparation 2 (direct injection).*

№	Compound	AREA				
		ST-2 S120122	ST-2 S140122	ST-2 S160122	ST-2 average	ST-2 sum
1	1,2,3-Benzotriazole	1,264,719	632,619	1,007,505	968,281	2,904,843
2	1,8-Diazabicyclo [5.4.0]undec-7-ene	-	-	-	-	-
3	10,11-Dihydro-10,11-dihydroxycarbamazepine	-	-	-	-	-
4	10-Hydroxycarbazepine	-	-	-	-	-
5	1-Naphthol	5,919,163	6,925,427	8,131,769	6,992,120	20,976,360
6	2,4-Diaminotoluene	-	-	-	-	-
7	2-Amino-4-cresol	431,040	245,481	458,947	378,489	1,135,468
8	2-Amino-6-methylmercaptopurine	346,843	275,835	452,725	358,468	1,075,403
9	2-Aminophenol/3-Hydroxy-2-methylpyridine/Nicotinyl alcohol	-	-	-	-	-
10	2-Methoxy-5-methylaniline	1,010,486	575,224	862,232	815,981	2,447,942
11	2-Methyl-S-benzothiazole	-	-	-	-	-
12	2-Phenylbenzimidazole-5-sulfonic acid (Ensulizole)	-	-	-	-	-
13	3,5-di-tert-Butyl-4-hydroxybenzoic acid	-	-	-	-	-
14	3-[(Hexanoyloxy)ethanimidoyl]-1H-pyrrole	-	-	-	-	-
15	4,4'-Dihydroxybiphenyl	-	-	-	-	-
16	4-Acetamidoantipyrine	3,189,779	2,618,239	3,178,767	2,995,595	8,986,785
17	4-Formylaminoantipyrine	-	-	-	-	-
18	4-Methylbenzotriazole	2,749,558	1,299,176	2,460,579	2,169,771	6,509,313
19	5-Methyl-1H-benzotriazole	2,145,723	457,773	736,180	1,113,225	3,339,676
20	6-Methoxyquinoline	-	-	-	-	-
21	6-Methyl-2-pyridinemethanol	455,630	460,579	693,743	536,651	1,609,952
22	Acetaminophen	-	-	-	-	-
23	Amantadine	-	-	-	-	-
24	Amisulpride	-	-	-	-	-
25	Amphetamine	-	-	-	-	-
26	Ampyrone	-	-	-	-	-
27	Articaine	7,311,859	7,836,363	15,512,495	10,220,239	30,660,717
28	Atenolol	459,235	419,032	527,355	468,541	1,405,622
29	Atenolol acid (Metoprolol acid)	1,383,297	938,337	1,052,347	1,124,660	3,373,981
30	Benzydamine	-	-	-	-	-
31	Betahistine	253,670	144,768	250,434	216,291	648,872
32	Bis(2-ethylhexyl) amine	-	-	-	-	-
33	Bisoprolol	509,500	341,028	2,417,095	1,089,207	3,267,622
34	Bupropion	-	-	-	-	-
35	Caffeine	-	-	-	-	-
36	Caprolactam	-	-	-	-	-
37	Carbamazepine	-	-	-	-	-
38	Chlorthiazide	-	-	-	-	-
39	Citalopram	583,775	505,246	585,473	558,165	1,674,494

№	Compound	AREA				
		ST-2 S120122	ST-2 S140122	ST-2 S160122	ST-2 average	ST-2 sum
40	Clarithromycin	-	-	-	-	-
41	Clindamycin	449,156	1,141,567	3,970,911	1,853,878	5,561,634
42	Clopidogrel carboxylic acid	-	-	-	-	-
43	Cordycepin	-	-	-	-	-
44	Darunavir	-	-	-	-	-
45	Decanamide	-	-	-	-	-
46	Decanophenone	167,715	473,871	-	213,862	641,586
47	DEET	-	-	-	-	-
48	Desacetyl diltiazem	-	-	-	-	-
49	Desmethylcitalopram	-	-	-	-	-
50	Dextromethorphan	-	-	-	-	-
51	Dibutyl adipate	-	-	-	-	-
52	Dibutyl hydrogen phosphate	-	-	-	-	-
53	Dibutyl phthalate	-	-	-	-	-
54	Diclofenac	-	-	-	-	-
55	Diethyl phthalate	-	-	-	-	-
56	Diltiazem	-	-	-	-	-
57	Diphenyl phosphate	-	-	-	-	-
58	Dodecyl sulfate	-	-	-	-	-
59	Dodecylbenzenesulfonic acid	-	-	-	-	-
60	Doxylamine	-	-	-	-	-
61	Erucamide	-	-	-	-	-
62	Esomeprazole	-	-	-	-	-
63	Fenofibric acid	-	-	-	-	-
64	Fexofenadine	-	-	-	-	-
65	Flecainide	684,909	610,016	699,414	664,779	1,994,338
66	Florfenicol	-	-	-	-	-
67	Fluconazole	-	-	-	-	-
68	Flufenamic acid	528,296	606,143	713,783	616,074	1,848,222
69	Gabapentin	-	-	-	-	-
70	Galaxolidone	1,310,690	1,615,180	1,054,573	1,326,814	3,980,443
71	Gemcitabine	-	-	-	-	-
72	Hexamethylenetetramine	6,686,485	4,955,323	5,935,703	5,859,170	17,577,511
73	Hydrochlorothiazide	411,114	308,639	356,609	358,787	1,076,362
74	Irbesartan	-	-	-	-	-
75	Ketamine	314,442	207,258	292,074	271,258	813,774
76	Ketoprofen	-	-	-	-	-
77	Lacosamide	-	-	-	-	-
78	Lamotrigine	3,805,272	3,515,892	4,251,726	3,857,630	11,572,891
79	Levamisole	-	-	-	-	-
80	Lidocaine	-	-	-	-	-
81	Lincomycin	-	-	-	-	-
82	Losartan	-	-	-	-	-
83	Melamine	-	-	-	-	-
84	Mepivacaine	-	-	-	-	-

№	Compound	AREA				
		ST-2 S120122	ST-2 S140122	ST-2 S160122	ST-2 average	ST-2 sum
85	Metformin	1,645,571	1,924,908	5,695,429	3,088,636	9,265,908
86	Methocarbamol	-	-	-	-	-
87	Mirtazapine	160,272	127,428	138,441	142,047	426,141
88	m-Xylene-4-sulfonic acid (2,4-Dimethylbenzenesulfonic acid)	126,777	102,578	191,062	140,139	420,416
89	N,N'-Diphenylguanidine (DPG)	-	-	-	-	-
90	N-[(S)-(+)-1-Ethoxycarbonyl-3-phenylpropyl]-L-alanine	-	-	-	-	-
91	N-Butylbenzenesulfonamide	-	-	-	-	-
92	N-Ethylaniline	-	-	-	-	-
93	N-Ethyl-N-methylcathinone	-	-	-	-	-
94	Noramidopyrine	292,810	6,937,361	3,428,266	3,552,812	10,658,437
95	O-Desmethyl venlafaxine	-	-	-	-	-
96	Ofloxacin / Levofloxacin	-	-	-	-	-
97	Omeprazole sulfone	-	-	-	-	-
98	Oxcarbazepine	-	-	-	-	-
99	Perfluorobutanesulfonic acid (PFBS)	395,387	359,023	434,789	396,400	1,189,199
100	Perfluorohexanoic acid (PFHxA)	-	-	-	-	-
101	Phenazone	807,531	482,843	1,565,221	951,865	2,855,595
102	Pilocarpine	-	-	-	-	-
103	Pirlimycin	71,775	88,079	372,759	177,538	532,613
104	Propranolol	-	-	-	-	-
105	Pyrimethanil	-	-	-	-	-
106	Pyroquilon	-	-	-	-	-
107	Sitagliptin	440,742	324,908	399,543	388,398	1,165,193
108	Sulfamethoxazole	-	-	-	-	-
109	Sulpiride	692,911	606,163	661,917	653,664	1,960,991
110	Tapentadol	1,369,407	1,059,033	1,246,471	1,224,970	3,674,911
111	Terbutryn	-	-	-	-	-
112	Tiamulin	77,366	668,796	573,740	439,967	1,319,901
113	Trazodone	-	-	-	-	-
114	Tributyl phosphate	650,273	1,821,173	1,035,424	1,168,957	3,506,870
115	Tributylamine	-	-	-	-	-
116	Triethyl phosphate	-	-	-	-	-
117	Trimethoprim	-	-	-	-	-
118	Tris(2-butoxyethyl) phosphate	1,761,336	1,895,220	1,687,690	1,781,415	5,344,246
119	Venlafaxine	-	-	-	-	-
	Metconazole-d6	6,422,024	6,174,262	5,923,697	6,173,328	18,519,983

Value “-” means compound no detected

Table S7. *Chromatographic peak area obtained for each compound in sample preparation 3 (online SPE).*

№	Compound	AREA				
		ST-3 S120122	ST-3 S140122	ST-3 S160122	ST-3 average	ST-3 sum
1	1,2,3-Benzotriazole	3,590,077	2,737,153	1,276,823	2,534,684	7,604,053
2	1,8-Diazabicyclo [5.4.0]undec-7-ene	759,653	1,455,270	1,699,584	1,304,836	3,914,507
3	10,11-Dihydro-10,11-dihydroxycarbamazepine	-	-	-	-	-
4	10-Hydroxycarbazepine	-	-	-	-	-
5	1-Naphthol	-	-	-	-	-
6	2,4-Diaminotoluene	5,082,782	4,246,015	-	3,109,599	9,328,797
7	2-Amino-4-cresol	2,963,823	2,441,132	4,606,439	3,337,131	10,011,394
8	2-Amino-6-methylmercaptopurine	-	-	-	-	-
9	2-Aminophenol / 3-Hydroxy-2-methylpyridine / Nicotinyl alcohol	3,338,370	3,068,876	350,554	2,252,600	6,757,800
10	2-Methoxy-5-methylaniline	508,664	453,748	-	320,804	962,412
11	2-Methyl-S-benzothiazole	1,166,622	996,659	1,642,442	1,268,574	3,805,723
12	2-Phenylbenzimidazole-5-sulfonic acid (Ensulizole)	-	-	-	-	-
13	3,5-di-tert-Butyl-4-hydroxybenzoic acid	-	-	-	-	-
14	3-[(Hexanoyloxy)ethanimidoyl]-1H-pyrrole	-	-	-	-	-
15	4,4'-Dihydroxybiphenyl	8,172,641	-	-	2,724,214	8,172,641
16	4-Acetamidoantipyrine	8,392,866	6,694,309	2,819,222	5,968,799	17,906,397
17	4-Formylaminoantipyrine	-	-	-	-	-
18	4-Methylbenzotriazole	9,151,675	6,832,241	5,640,066	7,207,994	21,623,981
19	5-Methyl-1H-benzotriazole	1,850,355	1,212,878	2,569,634	1,877,622	5,632,867
20	6-Methoxyquinoline	-	-	-	-	-
21	6-Methyl-2-pyridinemethanol	-	5,844,335	2,389,924	2,744,753	8,234,259
22	Acetaminophen	-	-	-	-	-
23	Amantadine	-	-	-	-	-
24	Amisulpride	1,047,591	812,084	879,381	913,019	2,739,056
25	Amphetamine	-	-	-	-	-
26	Ampyrone	-	-	-	-	-
27	Articaine	13,692,246	16,010,940	21,066,707	16,923,298	50,769,893
28	Atenolol	1,933,290	2,286,187	3,100,049	2,439,842	7,319,526
29	Atenolol acid (Metoprolol acid)	818,894	544,534	1,101,356	821,595	2,464,784
30	Benzydamine	-	-	-	-	-
31	Betahistine	232,040	146,693	137,575	172,103	516,308
32	Bis(2-ethylhexyl) amine	801,409	833,441	829,577	821,476	2,464,427
33	Bisoprolol	317,315	302,037	-	206,451	619,352
34	Bupropion	-	-	-	-	-
35	Caffeine	-	-	-	-	-
36	Caprolactam	-	-	-	-	-
37	Carbamazepine	991,424	826,436	917,110	911,657	2,734,970
38	Chlorthiazide	-	-	-	-	-
39	Citalopram	1,725,534	1,652,066	1,651,838	1,676,479	5,029,438
40	Clarithromycin	-	-	-	-	-

№	Compound	AREA				
		ST-3 S120122	ST-3 S140122	ST-3 S160122	ST-3 average	ST-3 sum
41	Clindamycin	1,464,700	5,268,708	11,055,999	5,929,802	17,789,407
42	Clopidogrel carboxylic acid	-	-	-	-	-
43	Cordycepin	-	-	-	-	-
44	Darunavir	-	-	-	-	-
45	Decanamide	-	-	-	-	-
46	Decanophenone	-	2,097,107	-	699,036	2,097,107
47	DEET	1,109,437	1,207,899	981,568	1,099,635	3,298,904
48	Desacetyl diltiazem	431,820	384,030	291,334	369,061	1,107,184
49	desmethylcitalopram	515,687	483,350	496,703	498,580	1,495,740
50	Dextromethorphan	-	-	-	-	-
51	Dibutyl adipate	-	-	-	-	-
52	dibutyl hydrogen phosphate	1,656,430	4,695,582	3,075,674	3,142,562	9,427,685
53	Dibutyl phthalate	146,184	128,410	113,217	129,270	387,811
54	Diclofenac	-	-	-	-	-
55	Diethyl phthalate	-	-	-	-	-
56	Diltiazem	310,186	319,107	291,266	306,853	920,559
57	Diphenyl phosphate	-	-	-	-	-
58	Dodecyl sulfate	-	-	-	-	-
59	Dodecylbenzenesulfonic acid	-	-	-	-	-
60	Doxylamine	908,164	973,711	1,160,274	1,014,050	3,042,149
61	Erucamide	-	-	-	-	-
62	Esomeprazole	-	-	-	-	-
63	Fenofibric acid	-	-	-	-	-
64	Fexofenadine	-	-	-	-	-
65	Flecainide	-	-	-	-	-
66	Florfenicol	-	-	-	-	-
67	Fluconazole	-	-	-	-	-
68	Flufenamic acid	1,012,148	1,254,945	1,483,544	1,250,212	3,750,637
69	Gabapentin	-	-	-	-	-
70	Galaxolidone	2,849,446	3,571,361	2,717,536	3,046,114	9,138,342
71	Gemcitabine	-	-	-	-	-
72	Hexamethylenetetramine	-	-	-	-	-
73	Hydrochlorothiazide	-	-	-	-	-
74	Irbesartan	691,534	879,027	1,203,910	924,824	2,774,471
75	Ketamine	-	-	-	-	-
76	Ketoprofen	-	-	-	-	-
77	Lacosamide	-	-	-	-	-
78	Lamotrigine	5,412,570	5,458,911	5,430,290	5,433,924	16,301,771
79	Levamisole	-	-	-	-	-
81	Lidocaine	-	-	-	-	-
82	Lincomycin	-	-	-	-	-
83	Losartan	1,235,198	1,235,198	1,248,282	1,239,559	3,718,678
84	Melamine	-	-	-	-	-
85	Mepivacaine	-	-	-	-	-
86	Metformin	4,596,524	6,150,920	13,970,304	8,239,249	24,717,748

№	Compound	AREA				
		ST-3 S120122	ST-3 S140122	ST-3 S160122	ST-3 average	ST-3 sum
87	Methocarbamol	-	-	-	-	-
88	Mirtazapine	-	-	-	-	-
89	m-Xylene-4-sulfonic acid (2,4-DIMETHYLBENZENESULFONIC ACID)	-	-	-	-	-
90	N,N'-Diphenylguanidine (DPG)	2,205,421	1,665,559	1,595,495	1,822,159	5,466,476
91	N-[(S)-(+)-1-Ethoxycarbonyl-3-phenylpropyl]-L-alanine	-	-	-	-	-
92	N-Butylbenzenesulfonamide	-	-	-	-	-
93	N-Ethylaniline	917,354	570,685	-	496,013	1,488,039
94	N-Ethyl-N-methylcathinone	-	-	-	-	-
95	Noramidopyrine	-	-	-	-	-
96	O-Desmethyl Venlafaxine	-	-	-	-	-
80	Ofloxacin / Levofloxacin	-	-	-	-	-
97	Omeprazole sulfone	-	-	-	-	-
98	Oxcarbazepine	454,183	580,997	459,587	498,255	1,494,766
99	Perfluorobutanesulfonic acid (PFBS)	-	-	-	-	-
100	Perfluorohexanoic acid (PFHxA)	-	-	-	-	-
101	Phenazone	1,856,065	1,100,885	1,843,311	1,600,087	4,800,260
102	Pilocarpine	-	-	-	-	-
103	Pirlimycin	208,354	444,180	1,349,786	667,440	2,002,320
104	Propranolol	271,021	261,295	260,174	264,163	792,490
105	Pyrimethanil	-	-	-	-	-
106	Pyroquilon	-	-	-	-	-
107	Sitagliptin	1,899,242	1,707,702	1,656,913	1,754,619	5,263,856
108	Sulfamethoxazole	-	-	-	-	-
109	Sulpiride	1,421,074	1,395,935	1,644,003	1,487,004	4,461,012
110	Tapentadol	4,058,006	3,965,840	4,008,245	4,010,697	12,032,091
111	Terbutryn	649,217	493,716	721,907	621,614	1,864,841
112	Tiamulin	214,948	2,243,932	1,826,345	1,428,408	4,285,225
113	Trazodone	-	-	-	-	-
114	Tributyl phosphate	3,440,587	10,264,780	6,447,188	6,717,518	20,152,555
115	Tributylamine	-	-	-	-	-
116	Triethyl phosphate	-	-	-	-	-
117	Trimethoprim	-	-	-	-	-
118	Tris(2-butoxyethyl) phosphate	7,647,836	8,729,084	8,268,406	8,215,108	24,645,325
119	Venlafaxine	-	-	-	-	-
	Metconazole-d6	1,245,384	1,123,505	1,155,341	1,174,743	3,524,230

Value “-” means compound no detected

Table S8. *PNEC and physical-chemical properties of the compounds identified estimated with QSAR models using EPI Suite program.*

№	Compound	PNEC Freshwater (µg/L)	Henry's Law constant (atm * m3 /mol)	log Koc	log Kow	Biowin 2 (Non-linear model prediction)	Biowin 3 (ultimate biodegradation time)	Biowin 6 (MITI non-linear model prediction)
1	1,2,3-Benzotriazole	19	1.47E-07	3.00	1.44	0.7887	2.9359	0.3935
2	1,8-Diazabicyclo [5.4.0]undec-7-ene	21.2	1.93E-06	3.16	1.38	0.6999	2.8627	0.5057
3	10,11-Dihydro-10,11-dihydroxycarbamazepine	1.91	7.44E-16	1.00	-0.21	0.8032	2.9218	0.1439
4	10-hydroxycarbazepine	100	2.04E-14	2.29	0.93	0.7489	2.7223	0.0623
5	1-Naphthol	2.76	5.47E-08	3.48	2.85	0.8665	2.9369	0.4238
6	2,4-Diaminotoluene	12	7.43E-10	2.08	0.14	0.1231	2.5844	0.0581
7	2-Amino-4-cresol	40.2	2.19E-10	2.17	1.16	0.6982	2.7736	0.2497
8	2-Amino-6-methylmercaptopurine	0.49	2.82E-10	1.82	0.18	0.1867	2.6638	0.0220
9	2-Aminophenol /3-Hydroxy-2-methylpyridine/ Nicotinyl alcohol	4.04	1.98E-10	1.96	0.62	0.6132	2.8794	0.2927
10	2-Methoxy-5-methylaniline	23	1.24E-07	1.72	1.74	0.8786	2.6281	0.3427
11	2-Methyl-S-benzothiazole	0.69	1.09E-08	3.49	3.15	0.6070	2.7986	0.0991
12	2-Phenylbenzimidazole-5-sulfonic acid (Ensulizole)	100	1.31E-14	2.46	-0.16	0.4712	2.7572	0.0229
13	3,5-di-tert-Butyl-4-hydroxybenzoic acid	1.13	7.52E-11	3.02	4.36	0.3404	2.3660	0.3513
14	3-[(Hexanoyloxy)ethanimidoyl]-1H-pyrrole	0.56	1.62E-07	2.46	3.19	0.8450	3.0063	0.1450
15	4,4'-Dihydroxybiphenyl	1.19	4.48E-12	4.23	2.80	0.9817	2.9224	0.2866
16	4-Acetamidoantipyrine (N-Acetylaminoantipyrine)	100		2.38	-0.13	0.9823	2.6249	0.0470
17	4-Formylaminoantipyrine	1000	2.01E-11	1.80	0.50	0.9854	2.6559	0.0675
18	4-Methylbenzotriazole	5.9	1.62E-07	3.22	1.71	0.8449	2.8301	0.3428
19	5-Methyl-1H-benzotriazole	150	1.62E-07	3.21	1.71	0.8449	2.8301	0.3428
20	6-Methoxyquinoline	4.33	4.07E-08	3.12	2.37	0.9524	2.7893	0.4983

№	Compound	PNEC Freshwater (µg/L)	Henry's Law constant (atm * m3 /mol)	log Koc	log Kow	Biowin 2 (Non-linear model prediction)	Biowin 3 (ultimate biodegradation time)	Biowin 6 (MITI non-linear model prediction)
21	6-Methyl-2-pyridinemethanol	46	3.14E-10	1.40	0.43	0.7886	2.7980	0.5144
22	Acetaminophen (Paracetamol)	46	6.42E-13	1.79	0.46	0.9886	2.8673	0.5090
23	Amantadine	25	8.32E-06	2.59	2.44	0.5616	2.6772	0.3328
24	Amisulpride	140	2.12E-20	2.80	1.10	0.1934	1.8806	0.0029
25	Amphetamine	24.8	1.08E-06	3.05	1.76	0.9898	2.8720	0.2199
26	Ampyrone	1.6	2.85E-12	2.45	-0.07	0.9540	2.7965	0.0480
27	Articaine	4.02	2.51E-13	2.27	2.10	0.9994	2.6063	0.1992
28	Atenolol	150	1.37E-18	2.17	0.16	0.9991	2.6078	0.2349
29	Atenolol acid (Metoprolol acid)	47.7	6.71E-17	1.23	-2.34	0.9927	3.0245	0.2768
30	Benzydamine	0.15	3.21E-11	4.62	4.21	0.6081	2.2245	0.0256
31	Betahistine	23.7	2.33E-09	3.00	0.68	0.7547	2.6336	0.2066
32	Bis(2-ethylhexyl) amine	0.52	8.78E-04	4.78	6.56	0.9876	3.2867	0.3643
33	Bisoprolol	92	2.89E-15	1.52	1.87	0.0181	2.5889	0.0396
34	Bupropion	1.11	1.02E-07	3.01	3.85	0.0300	2.2526	0.0389
36	Caprolactam	67.4	2.53E-08	1.76	0.66	0.9836	2.8949	0.7904
37	Carbamazepine	2	1.08E-10	3.59	2.45	0.4143	2.6770	0.0364
38	Chlorthiazide	7.51	4.05E-12	2.03	-0.02	0.0389	2.3391	0.0022
39	Citalopram	16	2.69E-11	4.40	3.74	0.0000	1.5174	0.0001
40	Clarithromycin	0.12	1.73E-29	1.37	3.16	0.0000	1.2007	0.0000
41	Clindamycin	0.044	2.89E-22	1.77	2.16	0.0111	2.2491	0.0008
42	Clopidogrel carboxylic acid	0.65	6.92E-12	3.64	1.51	0.0124	2.3473	0.0049
43	Cordycepin	1.68	3.04E-21	1.00	-0.61	0.0251	2.8203	0.0283
44	Darunavir	0.22	5.39E-25	1.36	1.88	0.0001	1.8970	0.0000
45	Decanamide	2.52	1.08E-07	2.88	2.77	0.9940	3.0648	0.8724
46	Decanophenone	0.07	9.46E-05	3.81	5.60	0.9478	2.9835	0.6697
47	DEET	88	2.08E-08	2.73	2.18	0.9724	2.6474	0.3954
48	Desacetyl diltiazem	0.36	7.97E-16	3.03	1.29	0.8255	2.1689	0.0210
49	Desmethylcitalopram	0.5	1.23E-11	4.34	3.60	0.0000	1.8276	0.0003

№	Compound	PNEC Freshwater (µg/L)	Henry's Law constant (atm * m3 /mol)	log Koc	log Kow	Biowin 2 (Non-linear model prediction)	Biowin 3 (ultimate biodegradation time)	Biowin 6 (MITI non-linear model prediction)
50	Dextromethorphan	1.62	1.20E-07	3.95	3.60	0.1228	1.9995	0.0478
51	Dibutyl adipate	6.96	5.35E-06	2.54	4.33	1.0000	3.5053	0.9865
52	Dibutyl hydrogen phosphate	50.8	4.26E-09	2.18	2.29	0.9761	3.3313	0.2835
53	Dibutylphthalate	10	1.22E-06	3.16	4.51	1.0000	3.4612	0.9226
54	Diclofenac	0.05	4.73E-12	2.92	4.51	0.0000	1.1226	0.0000
55	Diethyl phthalate	73	3.94E-07	2.02	2.42	0.9997	2.9885	0.9153
56	Diltiazem	0.23	8.61E-17	3.98	2.70	0.9805	2.0562	0.0267
57	Diphenyl phosphate	28.1	1.06E-10	2.08	2.88	1.0000	2.3266	0.0027
58	Dodecyl sulfate	3.83	1.84E-07	4.01	2.42	0.7445	2.9088	0.3963
59	Dodecylbenzenesulfonic acid	0.12	6.27E-08	4.23	4.78	0.4415	2.8434	0.1749
60	Doxylamine	0.92	6.43E-12	4.11	2.37	0.0003	1.9339	0.0135
61	Erucamide	0.0071	2.84E-06	6.07	8.44	0.9399	2.6973	0.8135
62	Esomeprazole	100	3.04E-19	3.60	2.23	0.3710	1.4509	0.0000
63	Fenofibric acid	2.35	7.90E-12	2.45	4.00	0.0564	2.3600	0.0992
64	Fexofenadine	200	1.19E-18	0.82	2.81	0.0118	1.9801	0.0031
65	Flecainide	0.64	5.75E-13	4.70	3.78	0.0027	1.1116	0.0000
66	Florfenicol	7.81	7.30E-18	2.31	-0.04	0.1219	2.1670	0.0000
67	Fluconazole	0.25	1.02E-13	4.72	0.25	0.0000	1.4963	0.0000
68	Flufenamic acid	0.4	1.84E-10	3.08	5.25	0.0022	2.0176	0.0000
69	Gabapentin	1000	1.81E-10	1.77	-1.10	0.6472	2.9977	0.7053
70	Galaxolidone	0.1	6.59E-05	4.44	5.26	0.5868	2.2383	0.2030
71	Gemcitabine	5.7	1.70E-17	1.00	-2.01	0.0254	2.7167	0.0000
72	Hexamethylenetetramine	11	1.64E-09	1.74	-4.15	0.0004	1.8702	0.0304
73	Hydrochlorothiazide	100	4.39E-12	1.90	-0.07	0.0058	2.1997	0.0009
74	Irbesartan	700	4.15E-15	5.31	7.91	0.4342	2.2842	0.0042
75	Ketamine	5.71	1.38E-08	3.22	2.18	0.0308	2.2571	0.1032
76	Ketoprofen	2.1	2.12E-11	2.46	3.12	0.8770	2.9265	0.1848
77	Lacosamide	1000	3.25E-13	2.08	-0.16	0.9611	2.5510	0.1006
79	Levamisole	1.81	4.03E-10	3.94	1.84	0.8707	2.7697	0.1419

№	Compound	PNEC Freshwater (µg/L)	Henry's Law constant (atm * m3 /mol)	log Koc	log Kow	Biowin 2 (Non-linear model prediction)	Biowin 3 (ultimate biodegradation time)	Biowin 6 (MITI non-linear model prediction)
80	Lidocaine	600	1.31E-10	2.96	2.44	0.7864	2.2226	0.1311
81	Lincomycin	3.95	3.00E-23	1.77	0.56	0.2220	2.6230	0.0070
82	Losartan	78	4.35E-16	5.96	4.01	0.1864	2.4414	0.0006
83	Melamine	360	1.89E-13	1.32	-1.37	0.0000	2.2697	0.0000
84	Mepivacaine	3.49	7.67E-11	3.23	1.95	0.7564	2.1960	0.0613
85	Metformin	156	7.64E-16	2.15	-1.40	0.7640	2.9137	0.2379
86	Methocarbamol	24.9	6.53E-16	1.12	0.61	0.9980	2.6631	0.7086
87	Mirtazapine	1	1.06E-12	4.45	3.03	0.0019	1.8141	0.0038
88	m-Xylene-4-sulfonic acid (2,4-DIMETHYLBENZENESULFONIC ACID)	111	3.06E-09	1.50	-0.07	0.6201	2.7801	0.2319
89	N,N'-Diphenylguanidine (DPG)	1.05	7.12E-12	3.77	2.78	0.8455	2.6414	0.0266
90	N-[(S)-(+)-1-Ethoxycarbonyl-3-phenylpropyl]-L-alanine	5.33	2.94E-11	2.39	0.12	0.9993	3.0583	0.2558
91	N-Butylbenzenesulfonamide	21.1	2.17E-06	2.56	2.31	0.9740	3.0482	0.1514
92	N-Ethylaniline	0.81	5.55E-06	2.08	2.16	0.7649	2.8184	0.2406
93	N-Ethyl-N-methylcathinone	18	1.71E-07	2.47	2.55	0.3580	2.5212	0.1178
94	Noramidopyrine	21.1	6.27E-12	2.61	0.39	0.9444	2.7655	0.0332
95	O-Desmethyl Venlafaxine	0.006	3.59E-14	3.52	2.72	0.0395	2.1317	0.0668
96	Ofloxacin/ Levofloxacin	0.026	4.98E-20	1.65	-2.00	0.0000	1.5132	0.0001
97	Omeprazole sulfone	0.85	3.82E-17	3.68	1.58	0.8687	1.9204	0.0089
98	Oxcarbazepine	2.95	6.92E-13	3.68	1.58	0.3895	2.5443	0.0436
99	Perfluorobutanesulfonic acid (PFBS)	372	1.44E-05	2.26	2.41	0.0052	1.5793	0.0000
100	Perfluorohexanoic acid (PFHxA)	140	3.29E-03	2.35	3.48	0.0000	1.5083	0.0000
101	Phenazone	1.1	6.65E-10	2.35	0.38	0.8943	2.8052	0.0962
102	Pilocarpine	0.1	2.88E-07	2.19	0.12	0.9911	2.8043	0.3825
103	Pirlimycin	1.61	1.31E-22	1.67	1.80	0.2780	2.5593	0.0017
104	Propranolol	0.2	7.98E-13	3.09	3.48	0.9782	2.7523	0.2010
105	Pyrimethanil	1.5	2.46E-06	2.73	2.84	0.7730	2.4962	0.0424

№	Compound	PNEC Freshwater (μg/L)	Henry's Law constant (atm * m3 /mol)	log Koc	log Kow	Biowin 2 (Non-linear model prediction)	Biowin 3 (ultimate biodegradation time)	Biowin 6 (MITI non-linear model prediction)
106	Pyroquilon	14.9	1.41E-08	2.29	1.57	0.6622	2.7191	0.3127
107	Sitagliptin	84	2.35E-15	6.38	1.39	0.0000	0.4606	0.0000
108	Sulfamethoxazole	0.1	9.56E-13	3.19	3.19	0.1281	2.4297	0.0060
109	Sulpiride	4.09	1.53E-17	3.16	0.57	0.7062	2.0775	0.0159
110	Tapentadol	53	1.27E-09	4.26	3.57	0.2949	2.4368	0.0669
111	Terbutryn	0.34	9.09E-09	2.80	3.74	0.0000	1.9379	0.0000
112	Tiamulin	0.25	4.21E-16	4.68	4.75	0.0013	1.4946	0.0147
113	Trazodone	0.016	1.74E-15	4.69	3.21	0.0002	1.6612	0.0007
114	Tributyl phosphate	37	3.19E-06	3.28	4.00	1.0000	3.6594	0.4729
115	Tributylamine	88	1.60E-04	3.63	4.46	0.9755	3.4298	0.5643
116	Triethyl phosphate	1600	3.60E-08	1.68	0.80	1.0000	2.9504	0.4364
117	Trimethoprim	120	2.39E-14	2.96	0.91	0.9164	2.0385	0.0172
118	Tris(2-butoxyethyl) phosphate	24	1.20E-11	3.10	3.75	1.0000	3.3413	0.1587
119	Venlafaxine	0.006	2.04E-11	3.17	3.28	0.1139	1.9862	0.1009

Table S9. Individual and total scores for prioritization.

Rank	Compound	Score P (0-1)	Score M (0-1)	Score B (0-1)	Score extra (0, 1)	Score PNEC (0-2)	Score Area (0-2)	Total Score (0-8)
1	Clindamycin	1	1	0	0	2	2	6
2	Flufenamic acid	1	0.5	1	0	2	1.5	6
3	O-Desmethyl Venlafaxine	1	0.5	0	0	2	2	5.5
4	Tiamulin	1	0	1	0	2	1.5	5.5
5	Galaxolidone	0.5	0	1	0	2	2	5.5
6	Venlafaxine	1	0.5	0	0	2	1.5	5
7	2,4-Diaminotoluene	1	1	0	0	1	2	5
8	2-Amino-6-methylmercaptopurine	1	1	0	0	2	1	5
9	Diclofenac	1	1	1	0	2	0	5
10	Terbutryn	1	1	0	0	2	1	5
11	Decanophenone	0	0.5	1	0	2	1.5	5
12	Bis(2-ethylhexyl) amine	0	0	1	1	1.5	1.5	5
13	Hexamethylenetetramine	1	1	0	0	1	1.5	4.5
14	Ofloxacin/ Levofloxacin	1	1	0	0	2	0.5	4.5
15	Pirlimycin	1	1	0	0	1.5	1	4.5
16	Melamine	1	1	0	1	0	1.5	4.5
17	Perfluorobutanesulfonic acid (PFBS)	1	1	0	1	0	1.5	4.5
18	Articaine	0.5	1	0	0	1	2	4.5
19	1,2,3-Benzotriazole	0	1	0	1	0.5	2	4.5
20	Phenazone	0	1	0	0	1.5	2	4.5
21	Lamotrigine	1	0.5	0	0	1	2	4.5
22	N,N'-Diphenylguanidine (DPG)	0.5	0.5	0	0	1.5	2	4.5
23	4-Methylbenzotriazole	0	0.5	0	1	1	2	4.5
24	Dibutylphthalate	0	0.5	1	1	1	1	4.5
25	2-Methyl-S-benzothiazole	0	0.5	0	1	1.5	1.5	4.5
26	Clarithromycin	1	1	0	0	2	0	4
27	Darunavir	1	1	0	0	2	0	4
28	Florfenicol	1	1	0	0	1	1	4
29	3-[(Hexanoyloxy)ethanimidoyl]-1H-pyrrole	0	1	0	0	1.5	1.5	4
30	Carbamazepine	1	0.5	0	0	1.5	1	4
31	Desacetyl diltiazem	0.5	0.5	0	0	2	1	4
32	Diltiazem	0.5	0.5	0	0	2	1	4
33	Desmethylcitalopram	1	0	0	0	2	1	4
34	Flecainide	1	0	0	0	1.5	1.5	4
35	2-Aminophenol	0	1	0	0	1	2	4
36	Doxylamine	1	0	0	0	1.5	1.5	4
37	Sulpiride	0.5	0.5	0	0	1	1.5	3.5
38	Amisulpride	1	1	0	0	0	1.5	3.5
39	Bisoprolol	1	1	0	0	0	1.5	3.5
40	Gemcitabine	1	1	0	0	1	0.5	3.5

Rank	Compound	Score P (0-1)	Score M (0-1)	Score B (0-1)	Score extra (0, 1)	Score PNEC (0-2)	Score Area (0-2)	Total Score (0-8)
41	Lincomycin	1	1	0	0	1	0.5	3.5
42	2-Methoxy-5-methylaniline	0.5	1	0	0	0.5	1.5	3.5
43	4-Acetamidoantipyrine (N-Acetylaminoantipyrine)	0.5	1	0	0	0	2	3.5
44	Pyrimethanil	0.5	1	0	0	1.5	0.5	3.5
45	2-Amino-4-cresol	0	1	0	0	0.5	2	3.5
46	Ampyrone	0	1	0	0	1.5	1	3.5
47	N-Ethylaniline	0	1	0	0	1.5	1	3.5
48	Pilocarpine	0	1	0	0	2	0.5	3.5
49	Bupropion	1	0.5	0	0	1.5	0.5	3.5
50	Oxcarbazepine	1	0.5	0	0	1	1	3.5
51	Sulfamethoxazole	1	0.5	0	0	2	0	3.5
52	1-Naphthol	0	0.5	0	0	1	2	3.5
53	5-Methyl-1H-benzotriazole	0	0.5	0	1	0	2	3.5
54	Tapentadol	1	0	0	0	0.5	2	3.5
55	Atenolol	0.5	1	0	0	0	2	3.5
56	6-Methyl-2-pyridinemethanol	0	1	0	0	0.5	2	3.5
57	Dibutyl hydrogen phosphate	0	1	0	0	0.5	2	3.5
58	Propranolol	0	0.5	0	0	2	1	3.5
59	Irbesartan	1	0	1	0	0	1.5	3.5
60	4,4'-Dihydroxybiphenyl	0	0	0	0	1.5	2	3.5
61	Hydrochlorothiazide	1	1	0	0	0	1	3
62	Atenolol acid (Metoprolol acid)	0	1	0	0	0.5	1.5	3
63	Noramidopyrine	0	1	0	0	0.5	1.5	3
64	Ketamine	1	0.5	0	0	1	0.5	3
65	Mepivacaine	0.5	0.5	0	0	1	1	3
66	Mirtazapine	1	0	0	0	1.5	0.5	3
67	Chlorthiazide	1	1	0	0	1	0	3
68	Fenofibric acid	1	1	0	0	1	0	3
69	N-Ethyl-N-methylcathinone	1	1	0	0	0.5	0.5	3
70	Perfluorohexanoic acid (PFHxA)	1	1	0	1	0	0	3
71	DEET	0.5	1	0	0	0	1.5	3
72	Pyroquilon	0.5	1	0	0	1	0.5	3
73	10,11-Dihydro-10,11-dihydroxycarbamazepine	0	1	0	0	1.5	0.5	3
74	Ketoprofen	0	1	0	0	1.5	0.5	3
75	Metformin	0	1	0	0	0	2	3
76	3,5-di-tert-Butyl-4-hydroxybenzoic acid	1	0.5	0	0	1.5	0	3
77	Clopidogrel carboxylic acid	1	0.5	0	0	1.5	0	3
78	Dextromethorphan	1	0.5	0	0	1.5	0	3
79	Tributyl phosphate	0	0.5	0	0	0.5	2	3
80	Tris(2-butoxyethyl) phosphate	0	0.5	0	0	0.5	2	3
81	Citalopram	1	0	0	0	0.5	1.5	3

Rank	Compound	Score P (0-1)	Score M (0-1)	Score B (0-1)	Score extra (0, 1)	Score PNEC (0-2)	Score Area (0-2)	Total Score (0-8)
82	Fluconazole	1	0	0	0	2	0	3
83	Losartan	1	0	0	0	0.5	1.5	3
84	Sitagliptin	1	0	0	0	0.5	1.5	3
85	Trazodone	1	0	0	0	2	0	3
86	Benzydamine	0.5	0	0	0	2	0.5	3
87	Dodecyl sulfate	0	0	0	0	1	2	3
88	Dodecylbenzenesulfonic acid	0	0	1	0	2	0	3
89	Erucamide	0	0	1	0	2	0	3
90	Betahistine	0.5	1	0	0	0.5	1	3
91	Lidocaine	0.5	1	0	0	0	1	2.5
92	4-Formylaminoantipyrine	0.5	1	0	0	0	1	2.5
93	Diphenyl phosphate	0.5	1	0	0	0.5	0.5	2.5
94	Caffeine	0	1	0	0	1.5	0	2.5
95	Caprolactam	0	1	0	0	0.5	1	2.5
96	Cordycepin	0	1	0	0	1.5	0	2.5
97	Diethyl phthalate	0	1	0	0	0.5	1	2.5
98	N-[(S)-(+)-1-Ethoxycarbonyl-3-phenylpropyl]-L-alanine	0	1	0	0	1	0.5	2.5
99	Omeprazole sulfone	0.5	0.5	0	0	1.5	0	2.5
100	1,8-Diazabicyclo [5.4.0]undec-7-ene	0	0.5	0	0	0.5	1.5	2.5
101	Fexofenadine	1	1	0	0	0	0	2
102	10-hydroxycarbazepine	0.5	1	0	0	0	0.5	2
103	Amantadine	0.5	1	0	0	0.5	0	2
104	Lacosamide	0.5	1	0	0	0	0.5	2
105	Trimethoprim	0.5	1	0	0	0	0.5	2
106	Acetaminophen (Paracetamol)	0	1	0	0	0.5	0.5	2
107	Decanamide	0	1	0	0	1	0	2
108	Dibutyl adipate	0	1	0	0	1	0	2
109	Gabapentin	0	1	0	0	0	1	2
110	Methocarbamol	0	1	0	0	0.5	0.5	2
111	m-Xylene-4-sulfonic acid	0	1	0	0	0	1	2
112	6-Methoxyquinoline	0	0.5	0	0	1	0.5	2
113	Levamisole	0	0.5	0	0	1.5	0	2
114	2-Phenylbenzimidazole-5-sulfonic acid (Ensulizole)	0	1	0	0	0	0.5	1.5
115	N-Butylbenzenesulfonamide	0	1	0	0	0.5	0	1.5
116	Triethyl phosphate	0	1	0	0	0	0.5	1.5
117	Esomeprazole	1	0.5	0	0	0	0	1.5
118	Amphetamine	0	0.5	0	0	0.5	0.5	1.5
119	Tributylamine	0	0.5	0	0	0	1	1.5

Abbreviations: P, persistence; M, mobility; B, bioaccumulation.

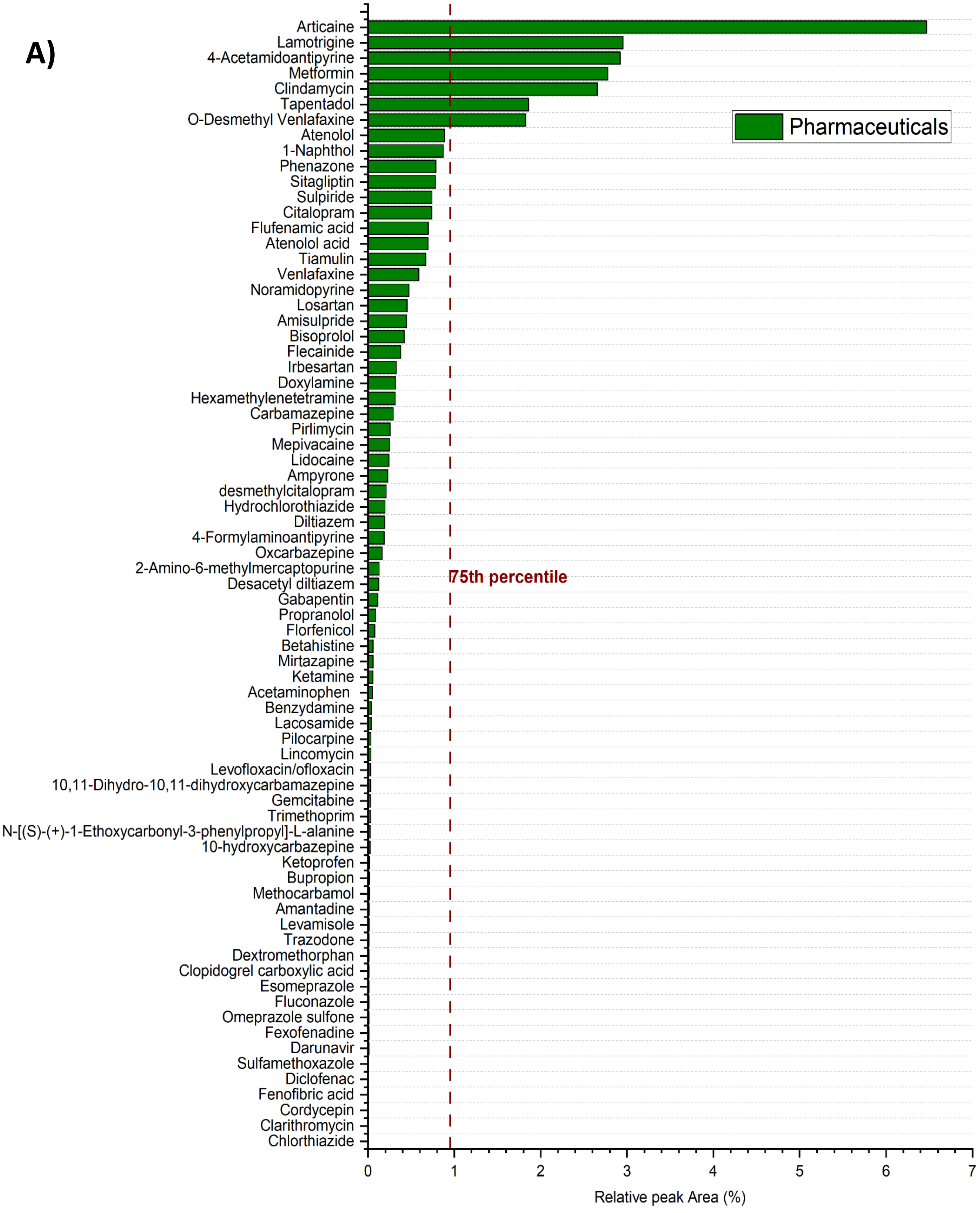
A)
Pharmaceuticals
75th percentile
Articaine
Lamotrigine
4-Acetamidoantipyrine
Metformin
Clindamycin
Tapentadol
O-Desmethyl Venlafaxine
Atenolol
1-Naphthol
Phenazone
Sitagliptin
Sulpiride
Citalopram
Flufenamic acid
Atenolol acid
Tiamulin
Venlafaxine
Noramidopyrine
Losartan
Amisulpride
Bisoprolol
Flecainide
Irbesartan
Doxylamine
Hexamethylenetetramine
Carbamazepine
Pirlimycin
Mepivacaine
Lidocaine
Ampyrone
desmethylcitalopram
Hydrochlorothiazide
Diltiazem
4-Formylaminoantipyrine
Oxcarbazepine
2-Amino-6-methylmercaptopurine
Desacetyl diltiazem
Gabapentin
Propranolol
Florfenicol
Betahistine
Mirtazapine
Ketamine
Acetaminophen
Benzydamine
Lacosamide
Pilocarpine
Lincomycin
Levofloxacin/ofloxacin
10,11-Dihydro-10,11-dihydroxycarbamazepine
Gemcitabine
Trimethoprim
N-[(S)-(+)-1-Ethoxycarbonyl-3-phenylpropyl]-L-alanine
10-hydroxycarbazepine
Ketoprofen
Bupropion
Methocarbamol
Amantadine
Levamisole
Trazodone
Dextromethorphan
Clopidogrel carboxylic acid
Esomeprazole
Fluconazole
Omeprazole sulfone
Fexofenadine
Darunavir
Sulfamethoxazole
Diclofenac
Fenofibric acid
Cordycepin
Clarithromycin
Chlorthiazide
0
1
2
3
4
5
6
7
Relative peak Area (%)

B)

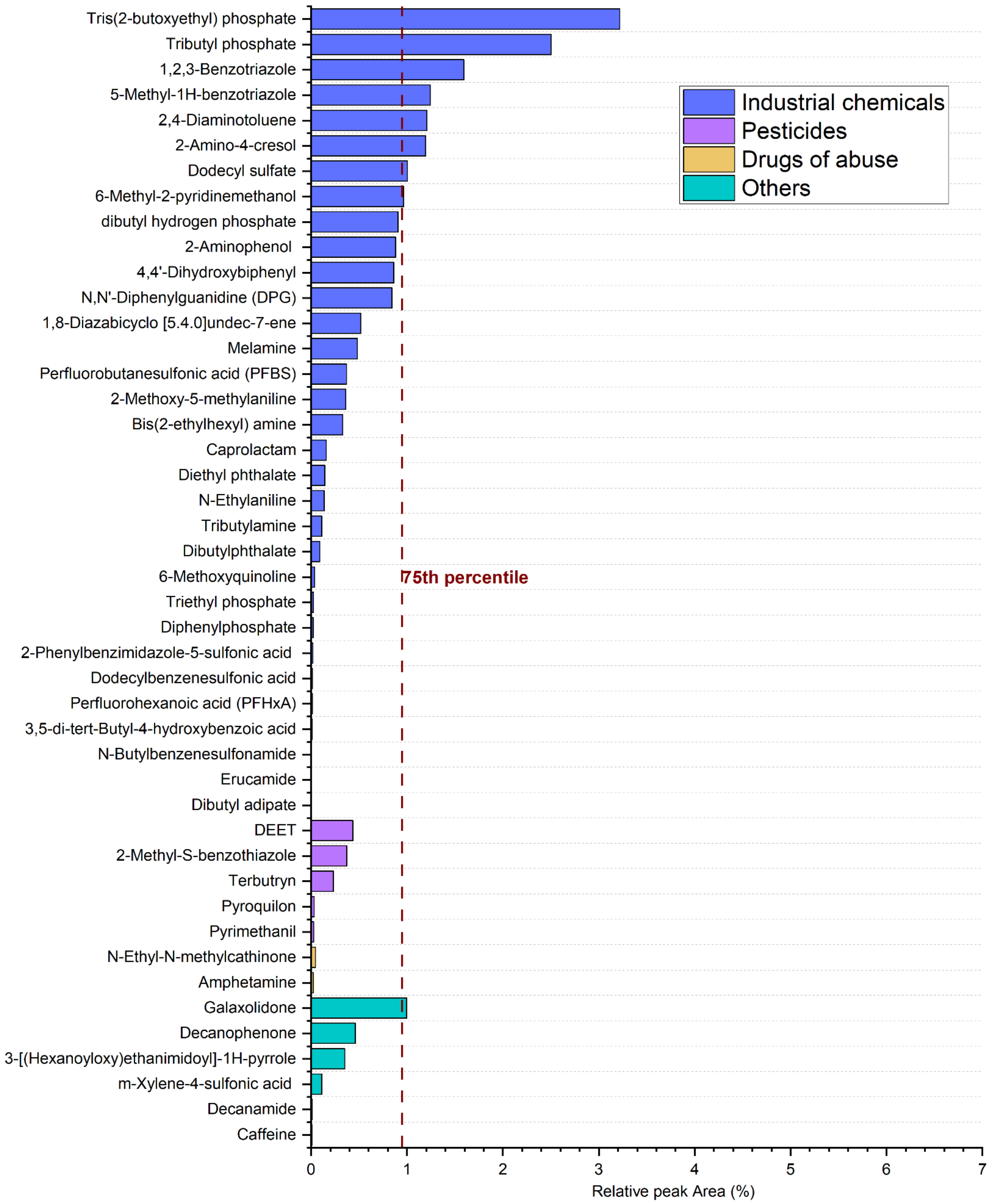

Fig. S1. *Relative peak area of A) pharmaceuticals and B) the remaining categories.*

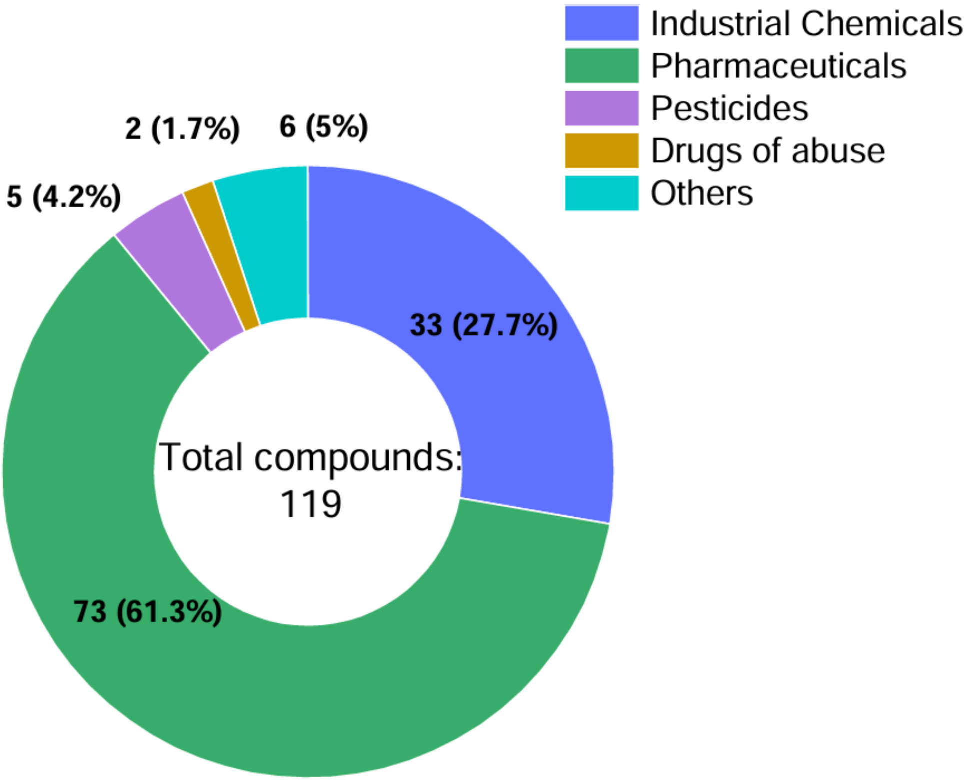

Fig. S2. *Percentage of suspect compounds identified in all samples per CEC category.*

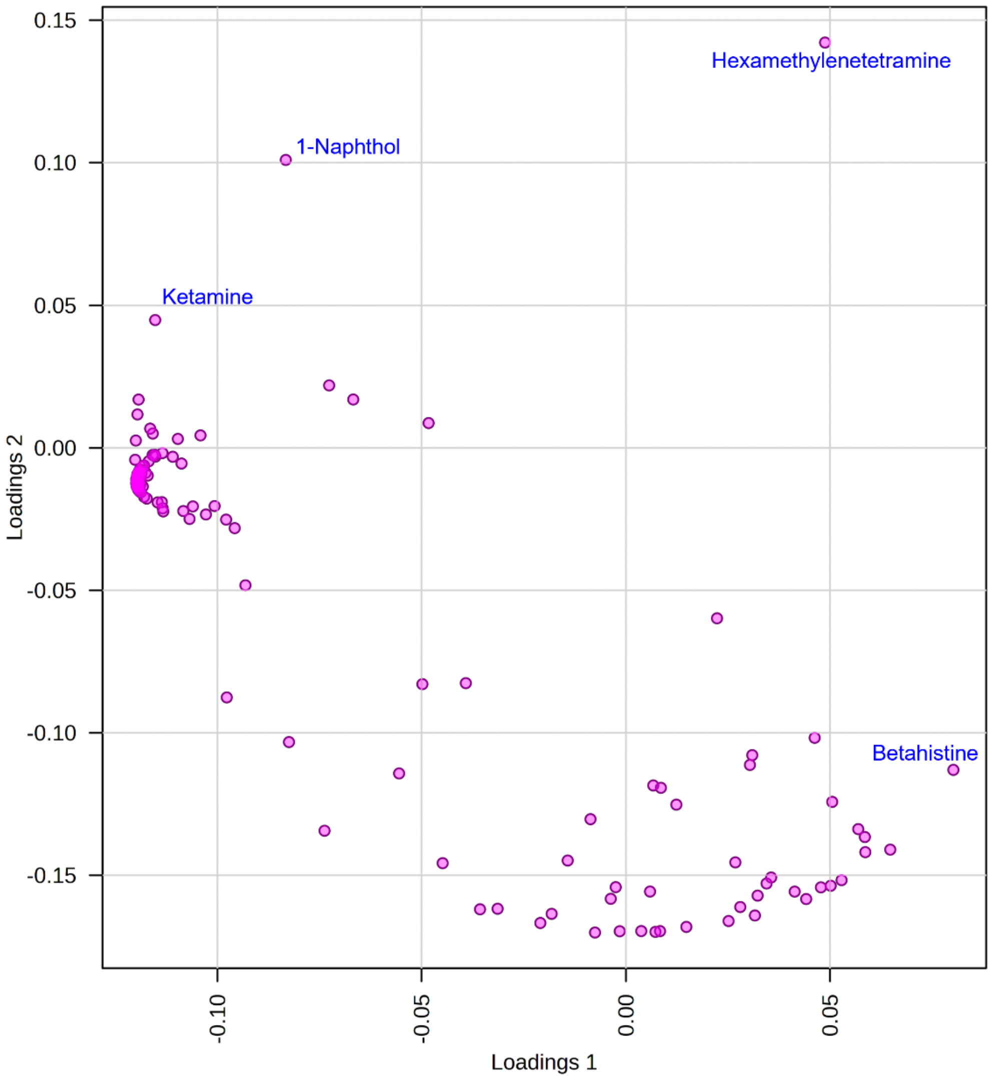

__Fig. S3__. PCA loading plot representing the variables (identified compounds) of the model.

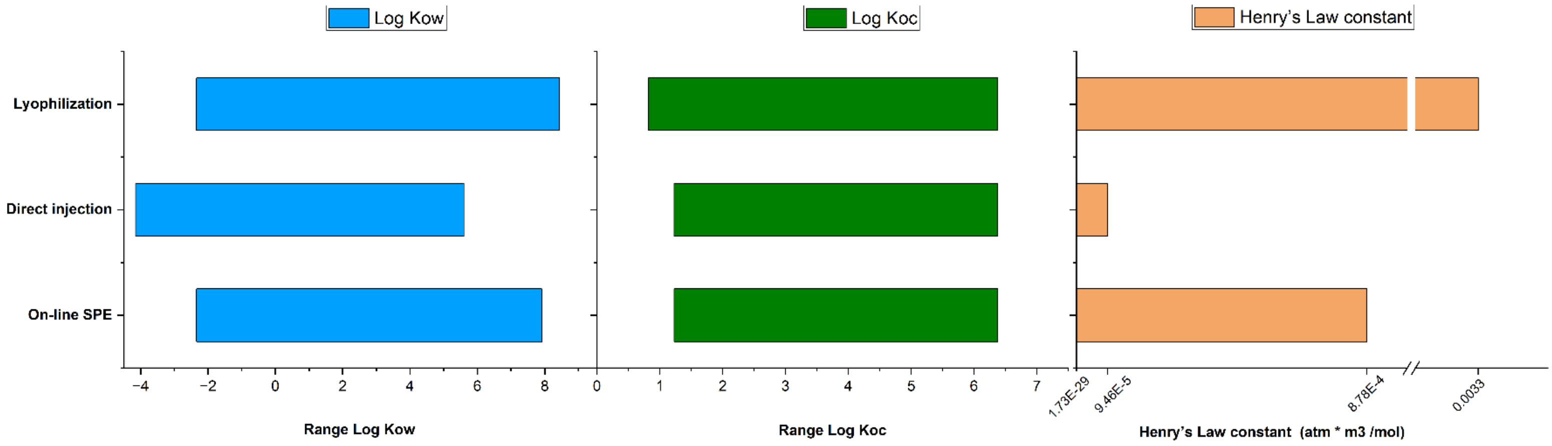

Fig. S4. *Range of n-octanol/water partition coefficients ($log\ K_{ow}$), organic carbon-water partition coefficients ($log\ K_{oc}$), and Henry's law constant of the compounds identified with each tested sample pretreatment procedure.*
